# Targeting the androgen receptor with siRNA promotes prostate cancer metastasis through enhanced macrophage recruitment via CCL2/CCR2-induced STAT3 activation

**DOI:** 10.1002/emmm.201202367

**Published:** 2013-08-27

**Authors:** Kouji Izumi, Lei-Ya Fang, Atsushi Mizokami, Mikio Namiki, Lei Li, Wen-Jye Lin, Chawnshang Chang

**Affiliations:** 1 George Whipple Lab for Cancer Research, Departments of Pathology, Urology, and Radiation Oncology, University of Rochester Medical CenterRochester, New York, USA; 2 Department of Integrative Cancer Therapy and Urology, Kanazawa UniversityKanazawa, Ishikawa, Japan; 3 Immunology Research Center, National Health Research InstitutesZhunan, Miaoli County, Taiwan; 4 Sex Hormone Research Center, China Medical University and HospitalTaichung, Taiwan

**Keywords:** androgen receptor, CCL2, epithelial–mesenchymal transition, prostate cancer, STAT3

## Abstract

Increased CCL2 expression in prostate cancer (PCa) cells enhanced metastasis via macrophage recruitment. However, its linkage to androgen receptor (AR)-mediated PCa progression remains unclear. Here, we identified a previously unrecognized regulation: targeting AR with siRNA in PCa cells increased macrophage recruitment via CCL2 up-regulation, which might then result in enhancing PCa invasiveness. Molecular mechanism dissection revealed that targeting PCa AR with siRNA promoted PCa cell migration/invasion via CCL2-dependent STAT3 activation and epithelial–mesenchymal transition (EMT) pathways. Importantly, pharmacologic interruption of the CCL2/CCR2-STAT3 axis suppressed EMT and PCa cell migration, providing a new mechanism linking CCL2 and EMT. Simultaneously targeting PCa AR with siRNA and the CCL2/CCR2-STAT3 axis resulted in better suppression of PCa growth and metastasis in a xenograft PCa mouse model. Human PCa tissue microarray analysis suggests that increased CCL2 expression may be potentially associated with poor prognosis of PCa patients. Together, these results may provide a novel therapeutic approach to better battle PCa progression and metastasis at the castration resistant stage via the combination of targeting AR with siRNA and anti-CCL2/CCR2-STAT3 signalling.

## INTRODUCTION

Prostate cancer (PCa) is the most common malignancy and the third leading cause of cancer death in males in the United States and many other countries (Siegel et al, 2011). Consistent with the pioneering study in 1941, androgen deprivation therapy (ADT) constitutes the major therapeutic approach to prevent androgens from binding to the androgen receptor (AR) as the basis to treat PCa (Huggins & Hodges, 1972). Questions have been raised about the central dogma regarding therapeutic efficacy of ADT for PCa since the curative effects of this ADT may become severely compromised when the PCa cells develop into a castration-resistant stage (Gulley et al, 2003). Previous studies have made tremendous efforts to understand the molecular mechanism of the failure of ADT, indicating the therapeutic approach that is solely based on targeting androgen/AR may be insufficient to control PCa cells (Harris et al, 2009; Kasper & Cookson, 2006; Yamaoka et al, 2010). Recently, two reports revealed a key mechanism of suppressing androgen/AR signalling that involves PI3K activation (Carver et al, 2011; Mulholland et al, 2011). Similarly, our previous study demonstrated that the genetic ablation of AR in prostate epithelial cells promoted the development of invasive PCa tumours (Niu et al, 2008), suggesting therapeutic suppressing androgen/AR function may elicit unwanted signals that may favour the progression of surviving PCa cells to the advanced stage.

Upon ADT treatments, we postulate that many PCa cells would be undergoing cell death via the therapeutically inhibited AR function, and dying PCa cells may prompt the recruitment of macrophages, which may provide a supportive microenvironment for the potential interaction between the macrophages and surviving PCa cells. Our previous study on molecular pathways linking AR function in macrophages and wound healing-associated inflammation showing that the deficit of AR in mice tends to create an immunosuppressive microenvironment that favours wound healing (Lai et al, 2009). These studies found a potential role for AR in mediating inflammatory responses during PCa progression since gene signatures of wound healing responses are very similar to genes identified in studies of progressive breast cancer with high metastatic potential (Chang et al, 2005).

Interestingly, one report showed that tumour-associated macrophages (TAMs) have been the major players to promote the development of hormonal resistance of PCa cells (Zhu et al, 2006), supporting a pro-tumour role for TAMs in the prostate tumour microenvironment. More importantly, Loberg et al used a xenograft model of PC3 cells to demonstrate that CCL2 may enhance prostate tumour growth/metastasis *in vivo* by increasing the recruitment of TAMs and angiogenesis (Loberg et al, 2007). This study highlights the important roles of CCL2 in directing infiltrating macrophages to enhance PCa progression/metastasis.

Similarly, it has been shown that castration-induced B cells infiltration and B cell-derived cytokines in PCa may play a key role in helping PCa cells become castration resistant (Ammirante et al, 2010). These results suggest a significant role for inflammatory cells in promoting castration resistance and metastasis of PCa cells. Nevertheless, the role of AR suppression in this regulation during ADT and its impact on the accompanying inflammation in this disease process has not been fully investigated. Hence, elucidating mechanisms by which suppressing androgen/AR results in activating downstream signalling pathways may have important implications for better therapeutic designs to control PCa progression instead of only targeting androgen/AR signalling.

In this study, we tested our hypothesis that suppressing AR function via siRNA in PCa might simultaneously trigger undesirable inflammatory signals that would prompt macrophage infiltration and thereafter could provide tumour-supporting signals to stimulate progression of PCa. We identified CCL2 as a key player in mediating STAT3 activation and epithelial–mesenchymal transition (EMT) of PCa cells and addressed the key problem of why targeting AR with siRNA might lead to promotion of PCa metastasis.

## RESULTS

### CCL2 is responsible for increased cell migration after targeting AR with siRNA in PCa and macrophages

To investigate the role of AR and mimic the crosstalk between macrophages and PCa cells in the tumour microenvironment, we established an *in vitro* co-culture model that allows the crosstalk between infiltrating macrophages and PCa cells in the presence or absence of AR silencing. We determined whether silencing macrophage AR function via lentiviral AR-siRNA (siAR) using scramble RNA (scr) as a control, would modulate behaviours of PCa cells during co-culture since we hypothesized that infiltrating macrophages could be increased during ADT and the macrophage function could possibly be affected by targeting AR with siAR. THP-1 cells have been characterized as M2-like macrophages and the AR ablation in myeloid cells tends to establish an immunosuppressive environment for wound healing (Kaler et al, 2009; Lai et al, 2009).

**Figure 1:**
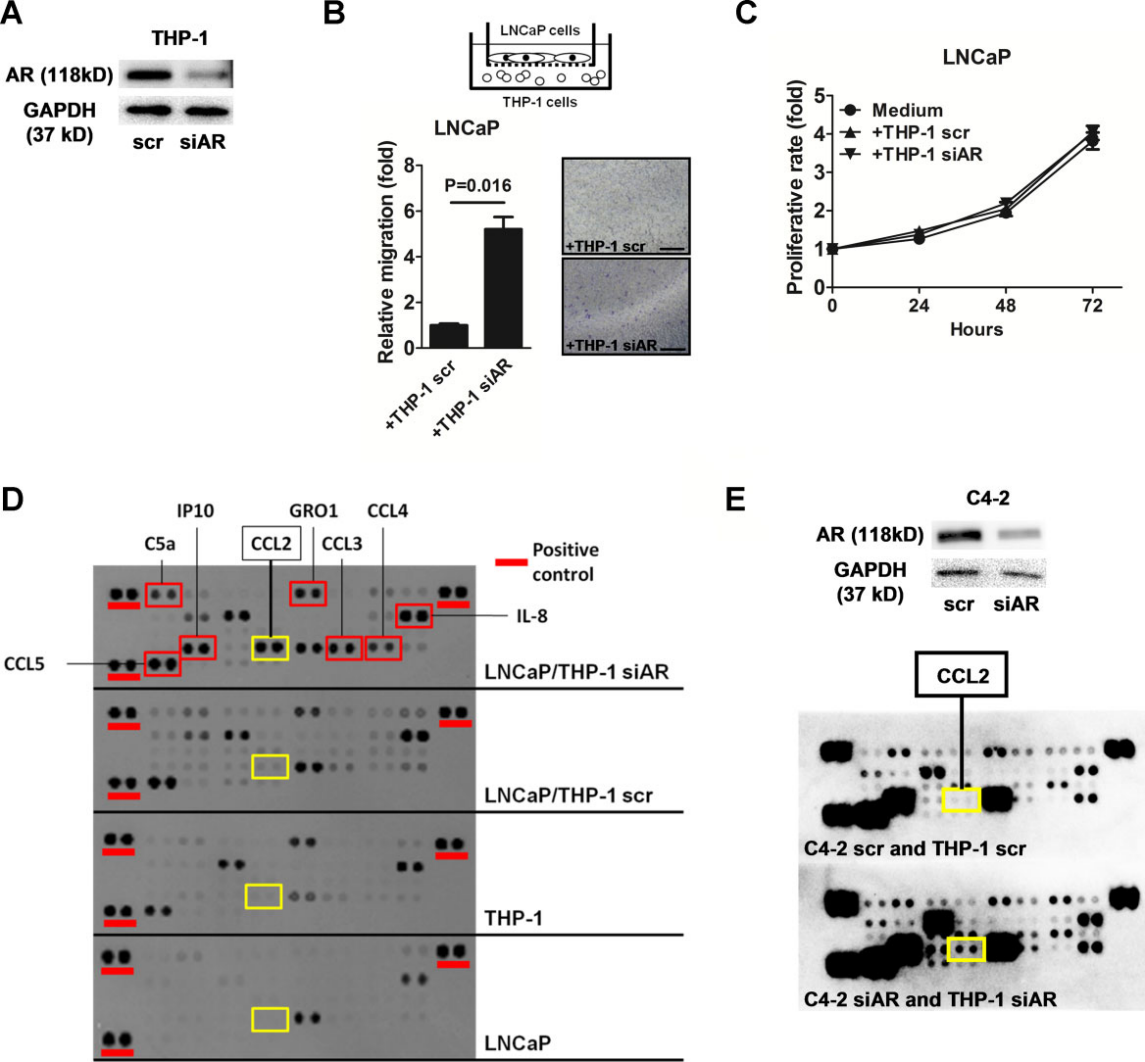
CCL2 is responsible for increased cell migration after targeting AR in macrophages and PCa cells Western blot of AR in THP-1 scramble (scr) and silenced AR (siAR) cells.Migration assay of LNCaP/THP-1 scr and LNCaP/THP-1 siAR cells co-cultured for 24 h. Schematic illustration of LNCaP/THP-1 co-culture is shown, (*n* = 3); *bar* in graph, Mean ± SEM; *bars* in pictures, 400 μm (magnification is 100×).Proliferation assay of LNCaP alone, LNCaP/THP-1 scr, or LNCaP/THP-1 siAR cells co-cultured for 24, 48 and 72 h, (*n* = 3).Cytokine array of different conditioned media (CM) of LNCaP and THP-1 cells. CM of LNCaP, THP-1, LNCaP/THP-1 scr and LNCaP/THP-1 siAR cells were collected after 24 h incubation. CCL2 showed the most obvious increase in co-cultured CM of LNCaP/THP-1 siAR among these four sets (yellow squares).Cytokine array of different CM of C4-2 and THP-1 cells. CM of C4-2 scr/THP-1 scr and C4-2 siAR/THP-1 siAR cells were collected after 24 h incubation. CCL2 showed obvious increase in co-cultured CM of C4-2 siAR/THP-1 siAR cells (yellow squares, lower panel). Western blot analysis of AR expression in C4-2 scr and siAR cells (upper panel).

We performed migration assays of LNCaP cells co-cultured with the macrophage cell lines, THP-1 scr and siAR cells (Fig 1**A**). The migration of LNCaP cells was significantly increased during co-culture with THP-1 siAR cells, as compared with THP-1 scr cells (Fig 1B). But, there was little effect on LNCaP proliferation during co-culture (Fig 1C). Next, we investigated whether AR silencing-induced pro-inflammatory cytokines were crucial players in mediating this crosstalk of enhanced LNCaP cell migration since early studies demonstrated that the co-culture of various types of cancer cells with macrophages might increase pro-inflammatory cytokines in the co-cultured conditioned medium (CM) (Alleva et al, 1994; Gleason et al, 1993; Said et al, 2007).

We first applied Western blot-based cytokine array analysis to globally identify inflammatory cytokines that could be important for mediating enhanced LNCaP cell migration in our co-culture system and found the most abundant cytokines/chemokines in the CM of THP-1 siAR and LNCaP cells were CCL2, CCL3, CCL4, GRO-1, CXCL10 (IP10) and C5a (Fig 1D).

To further mimic the suppressed AR signalling in the PCa microenvironment, we performed cytokine array analysis of the CM from co-culture of THP-1 and C4-2 cells with or without AR silencing in both macrophages and PCa cells. Consistently, we found targeting AR with siAR in both C4-2 cells and THP-1 cells increased expression of cytokines/chemokines, such as CCL2, IL-1ra, IL-16, CXCL11 and TNFα (Fig 1E). Among these increased cytokines by AR silencing, CCL2 has drawn our attention since early studies have shown CCL2 could promote cancer metastasis via recruitment of macrophages and the molecular mechanism of AR silencing-induced CCL2 expression remains elusive (Mizutani et al, 2009; Qian et al, 2011). Consistently, targeting AR with siAR in C4-2 cells alone increased only the expression of CCL2 (Supporting Information Fig S1), supporting a potential role for PCa cell-derived CCL2 in mediating local inflammatory responses when AR function is suppressed by siAR. We therefore hypothesized that induction of CCL2 by the interaction of infiltrated macrophages with surviving PCa cells during targeting AR via siAR might possibly obfuscate the benefits of anti-androgen/AR treatments, and may eventually facilitate the migration/invasiveness of the remaining PCa cells.

### Targeting PCa/macrophage AR with siAR leads to increased macrophage recruitment and enhanced PCa migration through CCL2 induction

**Figure 2:**
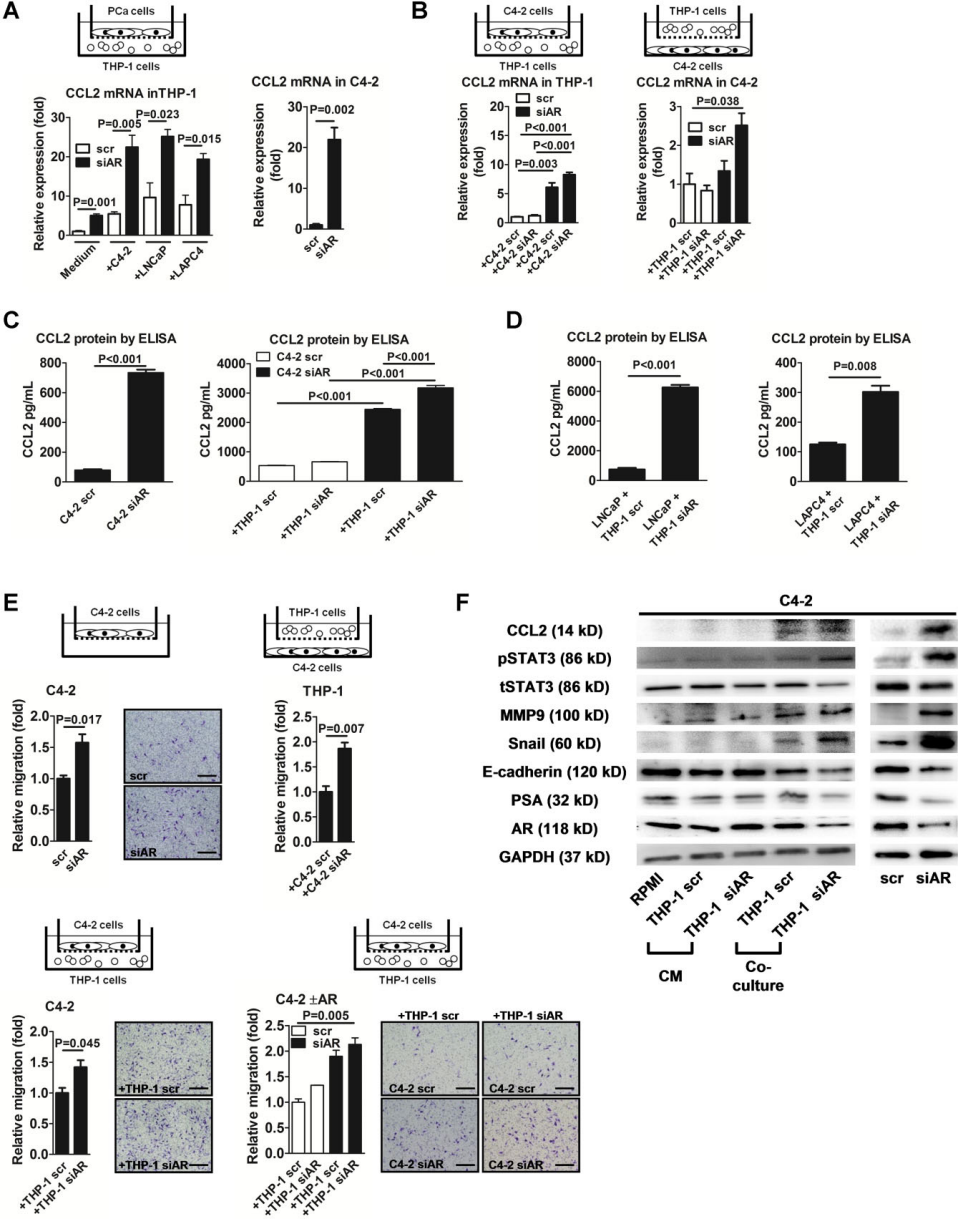
Targeting PCa/macrophage AR leads to increased macrophage recruitment and enhanced PCa migration through CCL2 induction qPCR of CCL2 mRNA in THP-1 scramble (scr) and THP-1 silenced AR (siAR) cells/different PCa cell lines as indicated (*left*) and qPCR of CCL2 mRNA in C4-2 scr and C4-2 siAR cells (*right*).qPCR of CCL2 mRNA in THP-1 (scr or siAR) cells co-cultured with C4-2 scr or siAR cells (*left*) and in C4-2 (scr or siAR) cells co-cultured with THP-1 scr or siAR cells (*right*).ELISA of CCL2 in 24 h CM of C4-2 scr and C4-2 siAR cells (*left*) and in 24 h co-cultured CM of C4-2 scr or C4-2 siAR cells/THP-1 scr or siAR cells (*right*).ELISA of CCL2 in 24 h co-cultured CM of parental LNCaP cells/THP-1 scr or siAR cells (*left*) and in 24 h co-cultured CM of parental LAPC4 cells/THP-1 scr or siAR cells (*right*).Migration assay of C4-2 scr and C4-2 siAR cells incubated for 24 h (*upper left*), parental THP-1 cells/C4-2 scr or siAR cells after co-cultured for 16 h (*upper right*), parental C4-2 cells/THP-1 scr cells or siAR cells after co-cultured for 24 h (*lower left*), and C4-2 scr or C4-2 siAR cells/THP-1 scr or siAR cells after co-cultured for 24 h (*lower right*), (*n* = 3); *bars* in graphs (A–E), Mean ± SEM; *bars* in pictures, 400 μm (magnification 100×).Western blot of CCL2, EMT markers, AR, and PSA in parental C4-2 cells treated with CM of THP-1 scr and siAR, or co-cultured with THP-1 scr and siAR cells for 24 h (*left*), and in C4-2 scr and siAR cells (*right*).

To determine whether the AR silencing-induced CCL2 expression in THP-1 cells could be further augmented during co-culture with other PCa cells (C4-2, LNCaP and LAPC4), we carried out quantitative real-time PCR (qPCR) and found CCL2 expression levels in THP-1 siAR cells were increased during co-culture with PCa cells (Fig 2A, *left*). Consistently, the expression levels of CCL2 were significantly increased in mono-cultured C4-2 siAR cells (Fig 2A, *right*). These results indicate that AR silencing via siAR in either macrophages or PCa cells may promote induction of CCL2. We also found that simultaneously silencing AR via siAR in both C4-2 and THP-1 cells can further augment CCL2 induction in THP-1 cells during co-culture (Fig 2B, *left*). Similarly, robustly increased CCL2 expression levels were observed in C4-2 siAR co-cultured with THP-1 siAR cells (Fig 2B, *right*). ELISA tests confirmed higher levels of CCL2 in the CM of C4-2 siAR cells (Fig 2C, *left*) and the highest levels of CCL2 in the CM of C4-2 siAR/THP-1 siAR cells (Fig 2C, *right*). Similar results were obtained from the CM of LNCaP or LAPC4 cells while co-cultured with THP-1 siAR cells (Fig 2D). From these experiments, we postulated that AR silencing via siAR in macrophages and PCa cells significantly enhanced induction of CCL2 via a positive feedback loop during co-culture.

We then determined whether AR silencing via siAR could also increase cell migration of PCa cells, since we observed increased CCL2 expression in AR silenced PCa cells and it has been shown that CCL2 controls PCa metastasis (Zhang et al, 2010b). We examined the cell migration of C4-2 cells and found C4-2 siAR cells have more migration capacity (Fig 2E, *upper left*). In addition, we examined if AR silenced PCa cells would increase THP-1 cell migration during co-culture, since we observed increased CCL2 in AR silenced PCa cells. Indeed, C4-2 siAR cells were able to recruit higher numbers of THP-1 cells (Fig 2E, *upper right*). Also, the number of migrated C4-2 cells was significantly increased when C4-2 cells were co-cultured with THP-1 siAR cells (Fig 2E, *lower left*). Similarly, more C4-2 siAR cells were able to migrate during co-culture with THP-1 siAR cells (Fig 2E, *lower right*). Importantly, THP-1 siAR cells skewed toward an M2-like phenotype with increasing M2 marker expression after co-culture with C4-2 cells (Sica et al, 2006) (Supporting Information Fig S2).

Taken together, these findings support our hypothesis that AR silencing via siAR in either THP-1 or C4-2 cells during co-culture could possibly enhance PCa cell migration or M2 polarization of THP-1 cells. We therefore reasoned that CCL2 up-regulation could be a potential player of this regulation.

We next investigated whether EMT and STAT3 activation is important for AR silencing-induced increased PCa cell migration since androgen deprivation has been linked to induction of EMT (Sun et al, 2012). EMT is thought to be an essential characteristic of cancer cells to invade and metastasize to a distant site (Friedl & Alexander, 2011). More importantly, STAT3 activation also has been reported to play an important role in inflammation, cancer progression and EMT induction (Abdulghani et al, 2008; Azare et al, 2007). We examined if the co-culture of THP-1 and C4-2 cells upon AR silencing via siAR would promote STAT3 activation and expression of EMT markers in C4-2 cells. Western blot analyses of phosphorylated STAT3 (pSTAT3), EMT markers (MMP9 and Snail), E-Cadherin, AR and PSA in C4-2 cells were performed. The mono-cultured CM derived from THP-1 cells did not have an impact on the expression of these markers, but the co-culture with THP-1 siAR increased expression levels of EMT markers and pSTAT3 (Fig 2F, *left*), consistent with the findings that show AR silencing via siAR in monoculture C4-2 cells promoted STAT3 activation and induction of EMT markers, as well as down-regulation of the epithelial marker, E-Cadherin (Fig 2F, *right*). Similar regulation was noted in LNCaP and LAPC4 cells co-cultured with THP-1 siAR cells (Supporting Information Figs S3 and S4).

Together, macrophage or prostatic epithelial AR silencing via siAR promotes STAT3 activation and EMT in PCa cells via induction of CCL2, which could possibly be associated with a secretory phenotype and pro-invasive characteristic of PCa cells.

### Neutralization of CCL2 inhibits migration, STAT3 activation, and induction of EMT in C4-2 cells

**Figure 3:**
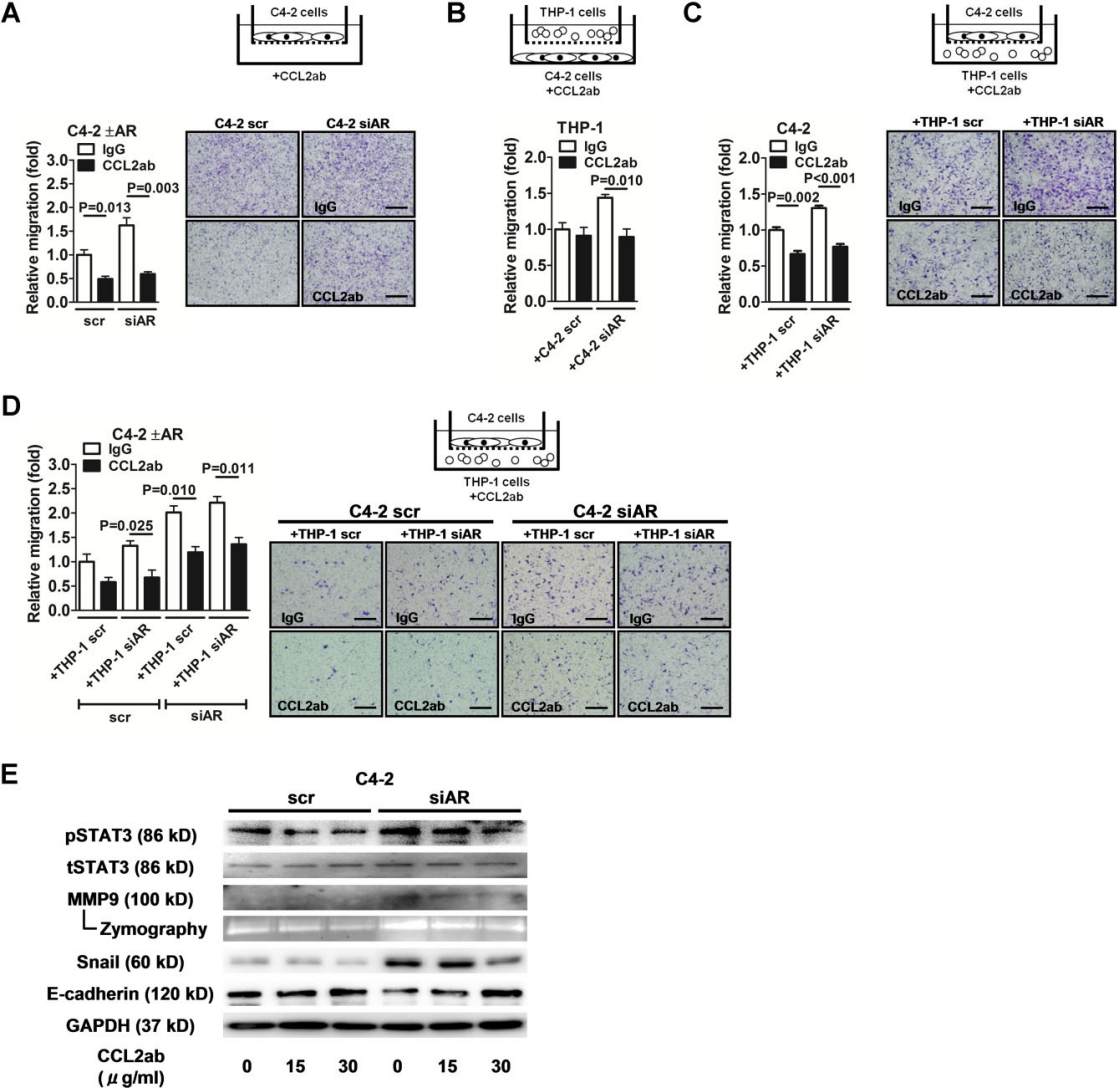
Neutralization of CCL2 inhibits migration, STAT3 activation and induction of EMT in C4-2 cells Neutralization of CCL2 in migration assay of C4-2 scramble (scr) and AR silenced (siAR) cells incubated for 24 h.Neutralization of CCL2 in migration assay of parental THP-1 cells + C4-2 scr or +C4-2 siAR cells co-cultured for 16 h.Neutralization of CCL2 in migration assay of parental C4-2 cells + THP-1 scr or +THP-1 siAR cells co-cultured for 24 h.Neutralization of CCL2 in migration assay of C4-2 scr and C4-2 siAR cells + THP-1 scr or +THP-1 siAR cells co-cultured for 24 h. Anti-CCL2 antibody (30 µg/ml; CCL2ab) and mouse IgG (control) were used in A–D. (*n* = 3); *bars* in graphs, Mean ± SEM in (A–D); *bars* in pictures, 400 μm (magnification 100×, A, C and D).Western blots of EMT markers (including the zymography of MMP9) in C4-2 scr and siAR cells incubated for 24 h with or without CCL2ab.

To determine whether induction of CCL2 by AR silencing via siAR in both cell types would be a crucial player in mediating cell migration, STAT3 activation and EMT in C4-2 cells, we examined whether inhibiting CCL2 activity by a neutralizing antibody would result in blocking migration of C4-2 cells. The neutralization of CCL2 by an anti-CCL2 antibody (CCL2ab) inhibited migration of C4-2 scr and siAR cells (Fig 3A). Similarly, CCL2ab inhibited migration of THP-1 cells during co-culture with C4-2 siAR cells (Fig 3B), and reduced migration of C4-2 cells that were co-cultured with THP-1 scr or siAR cells (Fig 3C). Consistently, CCL2ab inhibited migration of C4-2 scr and siAR cells that were co-cultured with either THP-1 scr or siAR cells (Fig 3D).

Together, these results suggest that, upon AR silencing via siAR, CCL2 is a key player in mediating the enhanced C4-2 cell migration and recruiting THP-1 cells during co-culture.

Next, we investigated if CCL2 is also an upstream signal that promotes STAT3 and EMT induction upon AR silencing via siAR. We found the protein levels of pSTAT3 in C4-2 scr and siAR cells were reduced by CCL2ab in a dose-dependent manner, as well as levels of EMT markers in C4-2 cells (Fig 3E), indicating CCL2 induction by AR silencing via siAR in PCa cells is an important upstream signal for STAT3 activation and EMT induction.

### AR silencing-induced CCL2/CCR2/STAT3 signalling controls EMT

**Figure 4:**
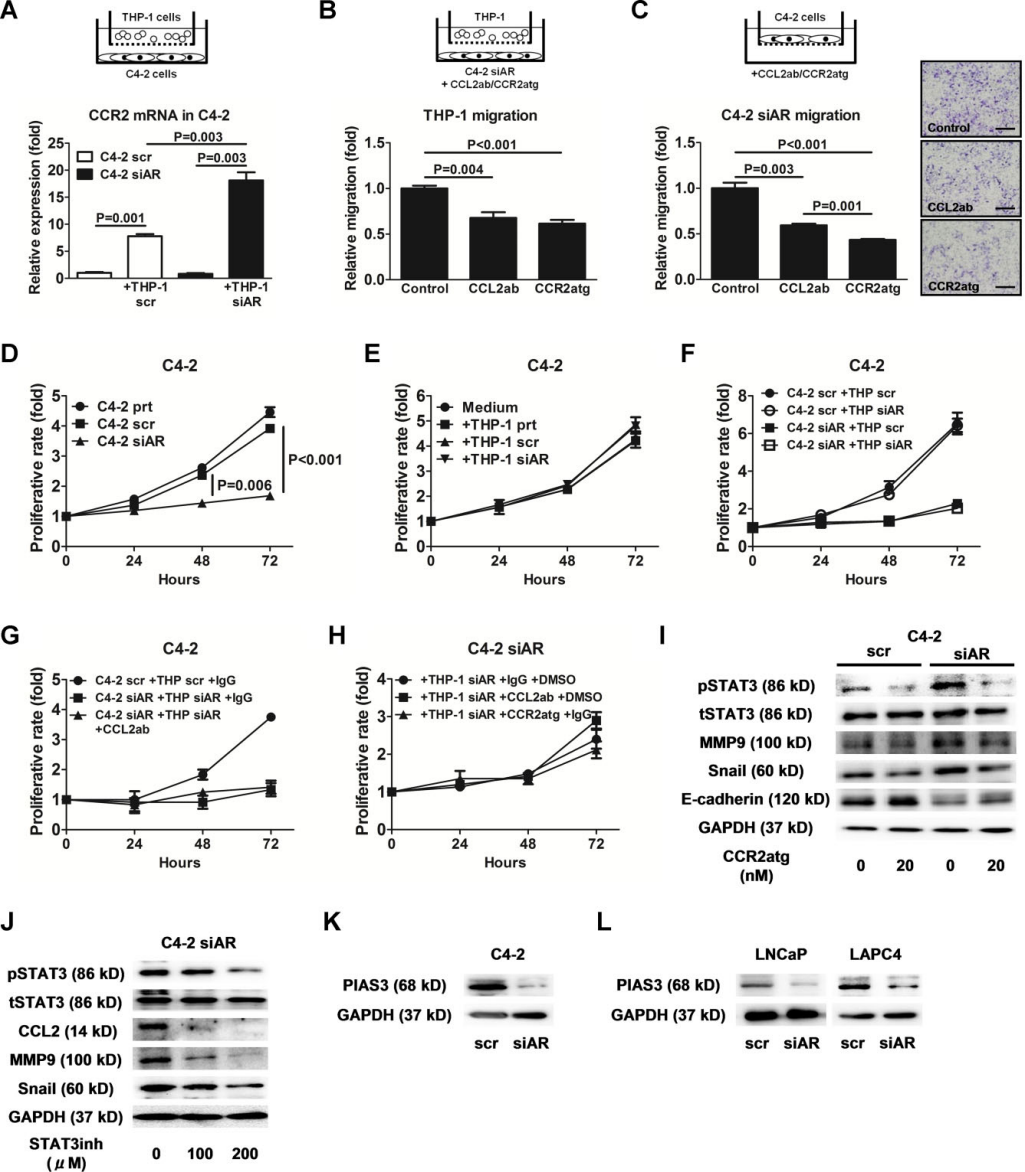
AR-silencing induced CCL2/CCR2/STAT3 signalling controls EMT qPCR of CCR2 in C4-2 scramble (scr) cells co-cultured with or without THP-1 scr cells and C4-2 AR silenced (siAR) cells co-cultured with or without THP-1 siAR cells for 24 h.Neutralization of CCR2 in migration assay of parental THP-1 cells + C4-2 siAR cells co-cultured for 16 h.Neutralization of CCR2 in migration assay of C4-2 siAR cells + THP-1 siAR cells co-cultured for 24 h. We used the same concentration of anti-CCL2 antibody (CCL2ab) in Fig 3 and 20 nM CCR2 antagonist (CCR2atg) diluted with DMSO used as treatment and DMSO used as control in (B and C), (*n* = 3); *bars* in graphs, Mean ± SEM in (A–C); *bars* in pictures, 400 μm (magnification 100×, C).Proliferation assay of parental C4-2, C4-2 scr and C4-2 siAR cells incubated for 24, 48 and 72 h.Proliferation assay of parental C4-2 cells + parental THP-1, +THP-1 scr, or +THP-1 siAR cells co-cultured for 24, 48 and 72 h.Proliferation assay of C4-2 scr and C4-2 siAR cells + THP-1 scr or +THP-1 siAR cells co-cultured for 24, 48 and 72 h.Neutralization of CCL2 in proliferation assay of C4-2 siAR cells + THP-1 siAR cells co-cultured for 24, 48 and 72 h. 30 µg/ml CCL2ab and mouse IgG (control) were used.Neutralization of CCR2 in proliferation assay of C4-2 siAR cells + THP-1 siAR cells co-cultured for 24, 48 and 72 h. 30 µg/ml CCL2ab and 20 nM CCR2atg diluted with DMSO were used as treatment, (*n* = 3); *bars* in graphs, Mean ± SEM in (D–H).Western blots of STAT3 and EMT markers in C4-2 scr and siAR cells incubated for 24 h with or without CCR2atg.Western blots of STAT3, CCL2 and EMT markers in C4-2 siAR cells incubated for 24 h with or without STAT3 inhibitor (STAT3inh).Western blot of PIAS3 in C4-2 scr and siAR cells.Western blot of PIAS3 in scr and siAR cells of LNCaP (*left*) and LAPC4 (*right*).

Subsequently, we determined whether prostatic CCR2 expression could be modulated by the crosstalk between AR silenced macrophages and PCa cells during co-culture. We speculate that AR silencing via siAR can potentially pre-condition PCa cells to respond to CCL2 by increasing CCR2, a specific receptor of CCL2 (Mizutani et al, 2009). Interestingly, CCR2 expression level in C4-2 siAR cells was much higher when co-cultured with THP-1 scr and siAR (Fig 4A), suggesting that the crosstalk between macrophages and PCa cells confer the robust increase of CCR2 in AR silenced PCa cells, which enable PCa cells to respond to CCL2 in the tumour microenvironment.

We also applied the second approach to interrupt CCL2/CCR2 signals by adding an antagonist of CCR2, CCR2atg, a novel di-substituted cyclohexane (Cherney et al, 2008; 2009). As expected, consistent with CCL2ab neutralization results, CCR2atg suppressed the migration ability of THP1 and C4-2 siAR cells (Fig 4B and C), confirming that CCL2 is the major player of AR silencing-induced chemotactic factors.

We next studied whether silencing AR via siAR in mono-cultured or co-cultured C4-2 cells would also modulate C4-2 cell proliferation. As expected, AR silencing via siAR in C4-2 significantly reduced C4-2 cell proliferation (Fig 4D), and the co-culture of C4-2 cells with AR silenced THP-1 cells did not further alter C4-2 cell proliferation (Fig 4E and F), suggesting CCL2 up-regulation did not play a role in regulating PCa cell proliferation. Moreover, neither treatments with CCL2ab nor CCR2atg had effects on C4-2 cell proliferation (Fig 4G and H). Importantly, inhibition of CCR2 by CCR2atg reduced expression levels of pSTAT3 and EMT markers (Fig 4I), suggesting that the CCL2/CCR2 axis plays a key role in promoting the AR silencing-mediated induction of EMT and STAT3 activation. Similarly, inhibition of STAT3 activity by a STAT3 inhibitor, AG490, resulted in down-regulation of EMT genes expression in C4-2 siAR cells (Fig 4J). This data suggests that STAT3 activation is essential for AR silencing-induced EMT. We next tested if STAT3 activation is required for CCL2 induction because we observed increased pSTAT3 protein levels in AR silenced PCa cells (Fig 4I), and it has been reported that STAT3 activates CCL2 promoter activity (Potula et al, 2009). Interestingly, AG490 also reduced AR silencing-induced CCL2 expression (Fig 4J). Taken together, these data all point to a reciprocal regulatory loop between CCL2 and STAT3 after AR is silenced via siAR in PCa cells.

To investigate the mechanisms of AR silencing-induced STAT3 activation in PCa cells, we investigated the protein inhibitor of STAT3, PIAS3 that is an AR-induced gene (Junicho et al, 2000). We found that silencing AR in various PCa cells dramatically reduced PIAS3 protein levels (Fig 4K and L), suggesting AR silencing in PCa cells might be able to function through down-regulation of PIAS3 to induce the STAT3 activation. Thus, our data demonstrated that the downstream target of AR silencing, CCL2, plays key roles to mediate THP-1 migration as well as PCa cell migration, and interruption of the CCL2/CCR2S/STAT3 axis with either anti-CCL2 antibody, CCR2 antagonist, or STAT3 inhibitor suppressed AR silencing-induced PCa cell migration and EMT induction. We concluded that CCL2/STAT3 play prominent roles in mediating EMT and cell migration in AR silenced PCa cells.

### Elimination of AR in mouse macrophages increases metastasis of TRAMP mice through induction of macrophage infiltration and CCL2

**Figure 5:**
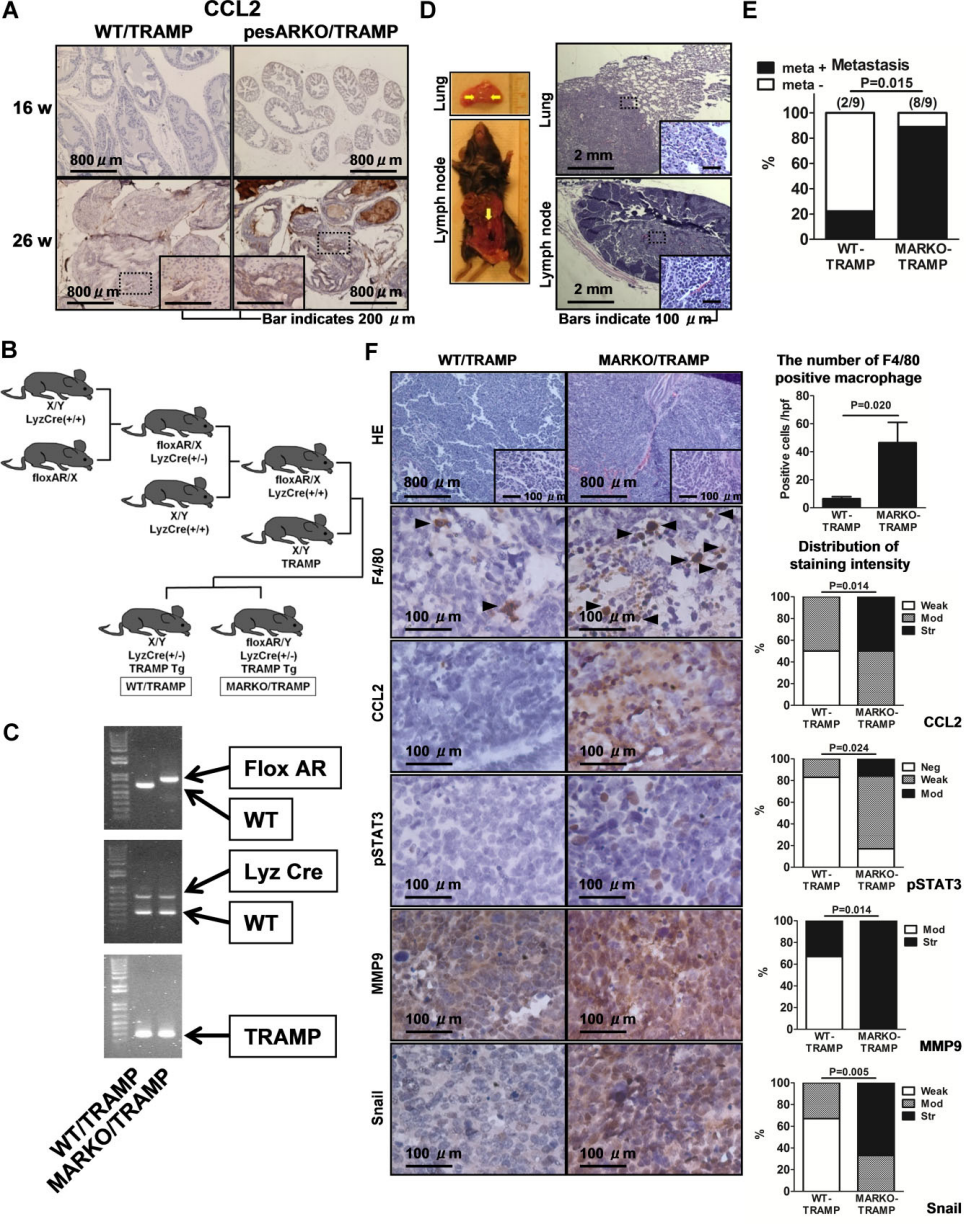
Elimination of AR in mouse macrophages increases metastasis of TRAMP mice through induction of macrophage infiltration and CCL2 IHC (magnification 400× and 100× for inset) staining of CCL2 in 16-week old WT/TRAMP and pesARKO/TRAMP mouse are shown.The breeding strategy to generate WT/TRAMP and MARKO/TRAMP mouse.WT/TRAMP and MARKO/TRAMP mice were confirmed by genotyping.Macroscopic photos (*left*) and haematoxylin eosin (H&E, magnification 40× and 400× for inset, *right*) staining of representative metastatic lesions in lung and lymph node of MARKO/TRAMP mouse are shown. Arrows indicate metastatic lesions.Statistical analysis of the number of metastases in WT/TRAMP and MARKO/TRAMP mouse. Graph shows the percentage of mice having metastasis (*n* = 9). Fisher's exact test was used.H&E (magnification 100× and 400× for inset) and IHC (magnification is 400×) staining of F4/80 (arrows indicate F4/80^+^ macrophages), CCL2, pSTAT3, MMP9, and Snail (*left*), and the distribution of staining intensity and statistical analysis (*right*). Chi-square test for trend was used, (*n* = 6); *bars* in graphs, Mean ± SEM.

We previously established a TRAMP mouse prostate tumour model with deletion of AR in prostate epithelial cells (pesARKO/TRAMP) and found this genetic ablation of AR unexpectedly increased metastasis of TRAMP prostate tumours (Niu et al, 2008), supporting a suppressive role for AR in PCa metastatic progression. We then examined CCL2 expression in the prostate tumour of pesARKO/TRAMP mice, and found increased CCL2 expression (Fig 5A).

We also examined the consequence of deletion of AR in macrophages on PCa development using a similar approach since our *in vitro* data demonstrated that AR silencing in THP-1 cells increased PCa cell migration and CCL2 expression (Fig 1B and D). We established the macrophage AR knockout TRAMP mouse (MARKO/TRAMP) model with wild type TRAMP mouse (WT/TRAMP) as control. Our breeding strategy is shown in Fig 5B and genotyping data are shown in Fig 5C. We found WT/TRAMP and MARKO/TRAMP mice were born at expected frequencies and the development of prostate gland remained normal. At around 28–32 weeks, we started to observe palpable tumours in MARKO/TRAMP mice. Two out of nine WT/TRAMP mice displayed metastasis in lung and lymph nodes (LN), but eight out of nine MARKO/TRAMP mice had metastasis (Fig 5D and E), suggesting that the ablation of AR in macrophages favours the development of metastatic prostate tumours in TRAMP mice.

Consistently, immunohistochemical (IHC) staining confirmed increased CCL2 expression in MARKO/TRAMP prostate tumours with increased numbers of F4/80 positive macrophages (Fig 5F). Importantly, we also found increased expression of EMT related genes such as pSTAT3, MMP9 and Snail in MARKO/TRAMP mice compared with those from WT/TRAMP mice (Fig 5F), suggesting that CCL2/STAT3/EMT axis could be the main driving force for metastasis.

Together, results from our *in vivo* MARKO/TRAMP mouse model confirm our *in vitro* cell lines studies showing AR silenced macrophages promote PCa metastasis through induction of CCL2 and macrophage infiltration.

### Combined targeting of PCa AR and anti-CCL2/CCR2 axis suppresses tumour growth and reduces metastasis in a xenograft mouse PCa model

**Figure 6:**
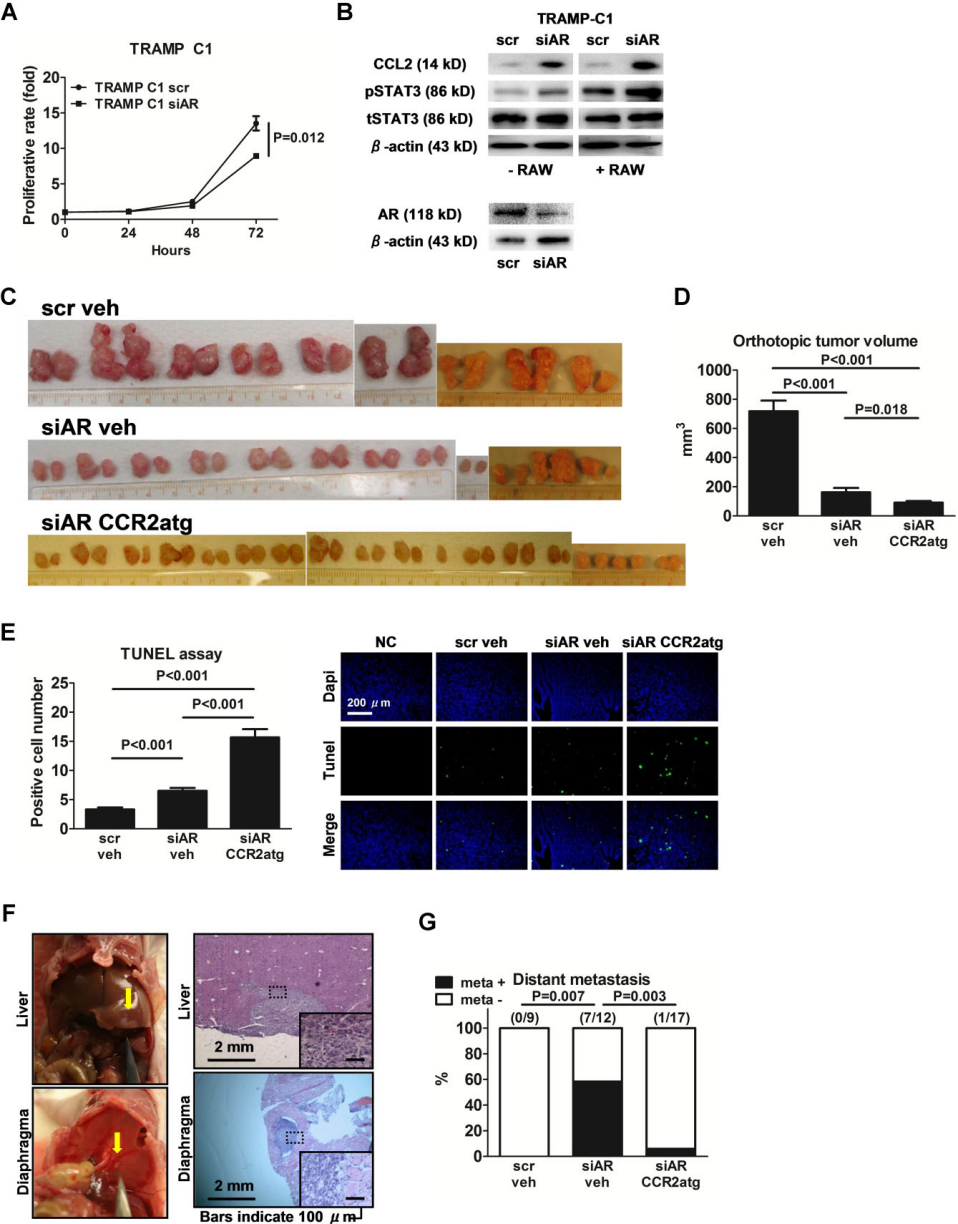
Combined targeting of PCa AR and CCL2/CCR2 axis suppresses tumour growth and reduces metastasis in a xenograft mouse PCa model Proliferation assay of TRAMP-C1 scramble (scr) and TRAMP-C1 AR silenced (siAR) cells incubated for 24, 48 and 72 h, (*n* = 3); *bars* in graphs, Mean ± SEM.Western blot of CCL2, STAT3 and AR in TRAMP-C1 scr and siAR cells co-cultured with or without RAW264.7 cells for 24 h.Macroscopic photos of orthotopic tumours in each group, TRAMP-C1 scramble + vehicle (scr veh), TRAMP-C1 siAR + vehicle (siAR veh), and TRAMP-C1 siAR + CCR2 antagonist (siAR CCR2atg) are shown.Quantification of tumour volume in each group (the number of tumours in scr veh, siAR veh and siAR CCR2atg is 18 from 9 mice, 24 from 12 mice, and 33 from 17 mice, respectively).Quantification of TUNEL assay in each group (*left*) and representative pictures (*right*) are shown (magnification is 100×). NC = negative control, (*n* = 6); *bars*, Mean ± SEM in (D and E).Macroscopic photos (*left*) and haematoxylin eosin (H&E, magnification 40× and 400× for inset, *right*) staining of representative metastatic lesions in liver and diaphragm of siAR veh mouse are shown. Arrows indicate metastatic lesions.Statistical analysis of the number of metastasis in scr veh, siAR veh and siAR CCR2atg mouse. Graph shows the percentage of the number of mice having metastasis. Fisher's exact test was used.

We first confirmed that AR silencing via siAR in mouse TRAMP-C1 cells inhibited cell proliferation, but increased expression of CCL2 and pSTAT3, and co-culture with mouse RAW264.7 cells resulted in further increased CCL2 and pSTAT3 expression (Fig 6A and B).

We then applied these mouse PCa cells and macrophages to test the contribution of AR and CCL2 to PCa progression *in vivo*. We orthotopically injected TRAMP-C1 cells (lentiviral scramble or siAR) into the anterior prostate lobes of nude mice. Importantly, during the development of palpable xenograft TRAMP-C1 tumours, mice were treated with CCR2atg or DMSO as vehicle control every other day. After treatment for 20 days, we found injection of DMSO or CCR2atg had little effect on mouse body weight. As expected, we observed reduced tumour volume of AR silenced TRAMP-C1 tumours (Fig 6C and D, scr vehicle vs. siAR vehicle, *p* < 0.001), confirming the AR function is essential for prostate tumour growth. Importantly, combined targeting of PCa AR (with AR-siRNA) and anti-CCL2/CCR2 axis (with CCR2atg) notably suppressed the growth of orthotopic TRAMP-C1 tumours (Fig 6C and D, siAR veh vs. siAR CCR2atg, *p* = 0.018). TUNEL assay also showed the orthotopic TRAMP-C1 siAR tumours + CCR2atg had the highest number of apoptotic cells (Fig 6E), suggesting that both AR and CCL2 pathways are essential signals for PCa tumourigenesis.

**Figure 7:**
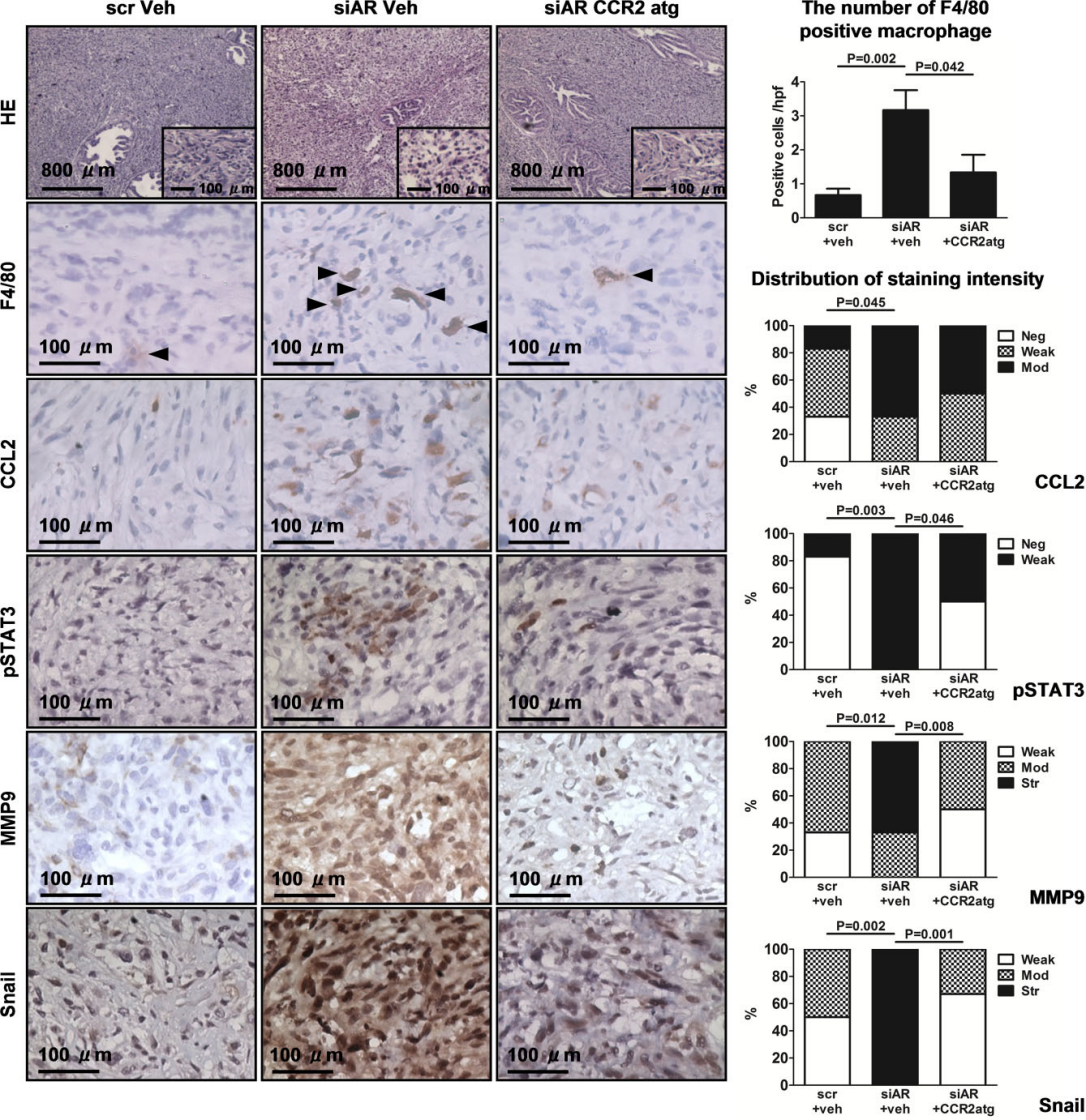
Increased EMT and macrophage recruitment in AR silenced tumours is mediated by the CCL2-STAT3 axis H&E (magnification 100× and 400× for inset) and IHC (magnification 400×) staining of F4/80 (arrows indicate F4/80^+^ macrophages), CCL2, pSTAT3, MMP9, Snail (*left*) and the distribution of staining intensity and statistical analysis (*right*). Chi-square test for trend was used. (*n* = 6), *bars* in graphs, Mean ± SEM.

Interestingly, although targeting PCa AR alone in TRAMP-C1 cells significantly reduced the tumour volume, we found mice with AR silenced TRAMP-C1 tumours had increased liver and diaphragm metastases (Fig 6F and G). Intriguingly, there was no difference among the number of LN metastases among these three groups. Thus, our results suggest that combined blockade of prostate AR and anti-CCL2/CCR2 signalling reduced primary tumour growth and distant metastases (Fig 6G, siAR veh vs. siAR CCR2atg, *p* = 0.003). IHC analysis confirmed markedly increased CCL2, pSTAT3, EMT markers (MMP9 and Snail) and F4/80-positive macrophages in TRAMP-C1 siAR tumours, and the treatment with CCR2atg significantly reduced these up-regulated markers (Fig 7). Consistently, the expression of PIAS3 was significantly low in TRAMP-C1 siAR tumours (Supporting Information Fig S5), confirming that PIAS3 is an AR downstream target, and the PIAS3 down-regulation by AR silencing could be an important step for STAT3 activation in PCa cells.

Together, these *in vivo* data confirm our *in vitro* data showing CCL2/CCR2/STAT3/EMT axis is an essential signalling pathway for AR silencing-mediated increased tumour metastasis, and provide new insights that combined targeting of both PCa AR and anti-CCL2/CCR2 axis may achieve the best therapeutic effects to suppress primary tumour PCa growth and metastasis.

### Increased CCL2 expression correlates with poor prognosis of PCa patients

**Figure 8:**
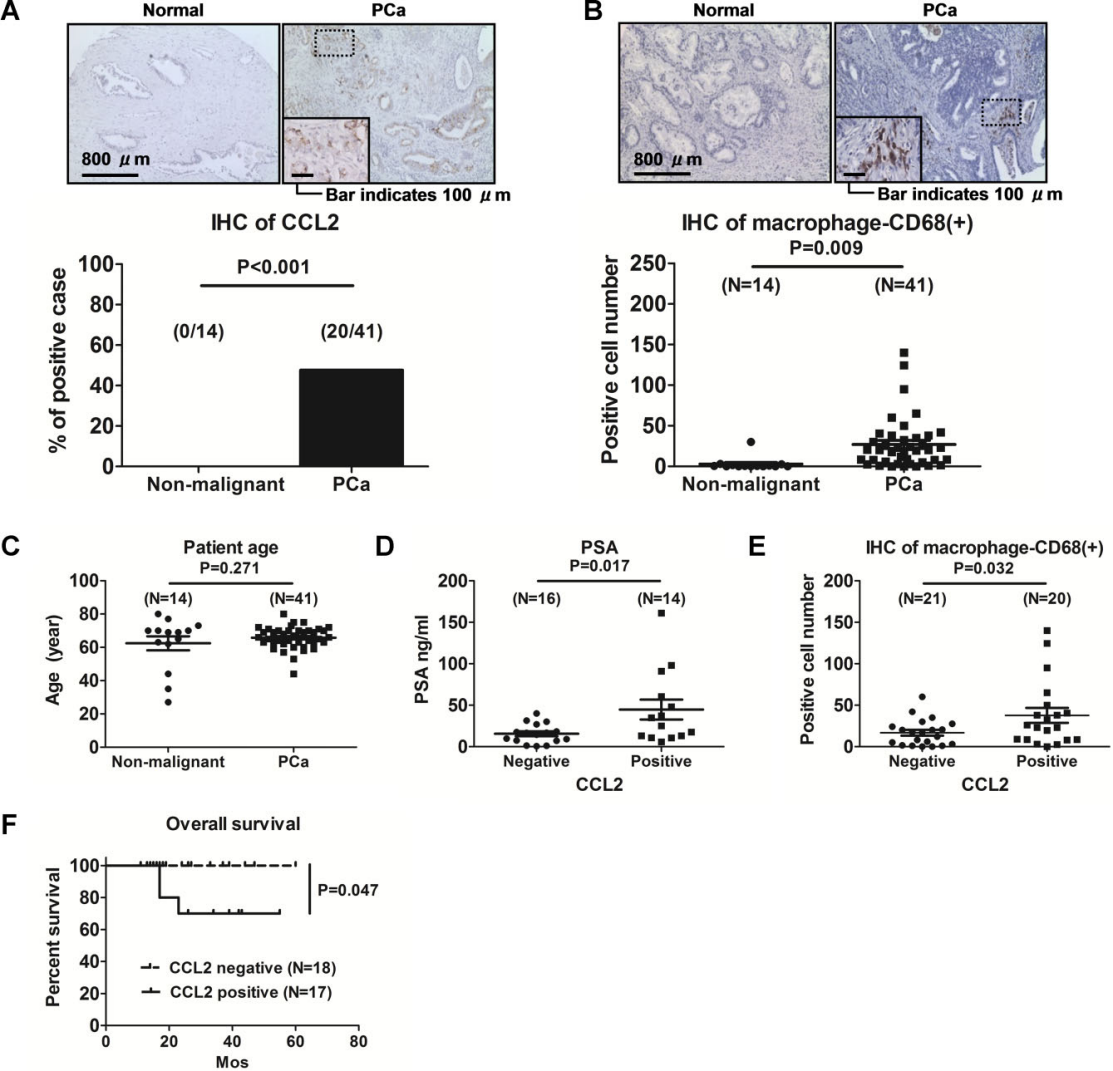
Increased CCL2 expression correlates with poor prognosis of PCa patients IHC of CCL2 in non-malignant prostate and PCa tissues, representative tissues are shown (*upper panels*, magnification 100× and 400× for inset). Fisher's exact test was used for % of positive cases (*lower panel*).IHC of CD68 in non-malignant prostate and PCa tissues, representative tissues are shown (*upper panels*, magnification 100× and 400× for inset). Positive cell number in each group are shown (*lower panel*); *bars*, Mean.Age background of non-malignant and PCa tissue is shown.The serum PSA values between prostate tissues from CCL2-negative and CCL2-positive PCa patients are compared.The number of CD68 positive cells in CCL2-negative and CCL2-positive PCa tissues are shown; *bars*, Mean ± SEM in (B–E).Overall survival curve of patients with tissue CCL2-negative and CCL2-positive tissues using Kaplan–Meier method is shown. Statistical analysis was done with Log-rank test.

We next extended our *in vitro* and *in vivo* findings to human PCa tissues, and attempted to establish the clinical significance of CCL2. We performed IHC analysis of the human prostate tissue microarray (TMA) that contains 14 benign prostate tissues and 41 primary PCa tissues, and found 20 out of 41 PCa samples were CCL2-positive. In contrast, no CCL2-positive signal was found in any of the 14 benign prostate samples (Fig 8A). Consistently, we also found more infiltrating CD68-positive macrophages in PCa as compared to benign prostate tissues (Fig 8B) and there were no age differences between these two groups (Fig 8C), suggesting a potential positive correlation of macrophages and CCL2 expression in human PCa tissues.

Interestingly, as we compared PSA values and CCL2 staining in 30 out of 41 PCa patients, we found that PSA value in CCL2-positive patients was significantly higher than those in CCL2-negative patients (Fig 8D), indicating CCL2 increase may be associated with PCa progression. Furthermore, tissue samples from CCL2-positive PCa patients had more macrophage infiltration than those from CCL2-negative PCa patients (Fig 8E), consistent with previous reports showing CCL2 promotes cancer progression via enhancement of macrophage recruitment (Qian et al, 2011; Zhang et al, 2010c). Most importantly, we found the outcome of PCa patients with CCL2-positive tissues was significantly worse with lower survival time than those PCa patients with CCL2-negative tissues (Fig 8F).

**Figure 9:**
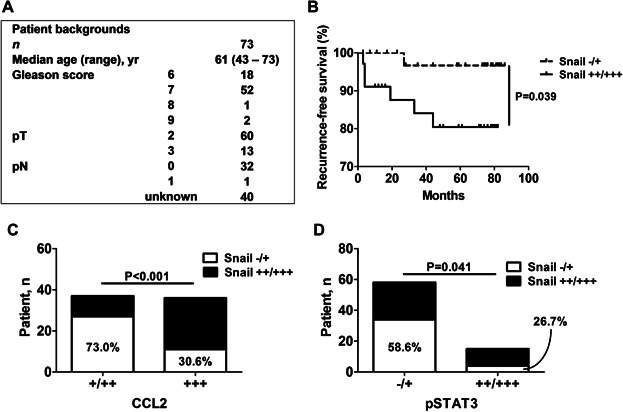
IHC analysis of CCL2, pSTAT3, snail in 73 PCa specimens The patient's information from radical prostatectomy is shown.Recurrence-free survival curve of patients with tissue snail-negative/-weakly positive and snail-moderately/-strongly positive tissues using Kaplan–Meier method is shown. Statistical analysis was done with Log-rank test.The association of Snail staining levels and CCL2 staining levels is shown.The association of snail staining levels and pSTAT3 staining levels is shown. Fisher's exact test was used in (C and D).

To further investigate whether increased expression of CCL2 downstream mediators, STAT3 and Snail, could possibly contribute to PCa progression, we performed IHC analysis of prostate TMAs containing 73 prostatectomy tissues (Fig 9A). Significantly, patient tissues with stronger Snail staining were correlated with poor recurrence-free survival (Fig 9B), and the expression levels of CCL2 and pSTAT3 are associated with Snail immune-reactivity in patient tissues (Fig 9C and D). This second set of human TMA analyses further confirms that CCL2/STAT3/Snail could be important markers with prognostic value, and targeting the CCL2/CCR2 axis may represent a potential new therapeutic approach to battle PCa, especially preventing the development of CRPC.

It remains unclear whether this CCL2-mediated pathway after AR blockade contributes to the development of CRPC, since this progression represents the major failure of ADT and shortens the survival of PCa patients (Garcia & Rini, 2012). We performed a pilot study by obtaining four pairs of PCa biopsy specimens that were collected at the time of diagnosis when patients were sensitive to ADT. Later, PCa specimens were re-biopsied from the same patients after confirming the diagnosis of CRPC. As the patient's information shows in Supporting Information Fig S6A, PSA values were significantly decreased after ADT. The number of macrophages increased after CRPC in 3 out of four patients in spite of their PSA decrease, and Case E had the highest number of macrophages (Supporting Information Fig S6B). In three out of four patients (Case A, C and D), CCL2 staining levels were increased after developing CRPC and no cases had CCL2 decrease after CRPC. Generally, the reduced expression level of AR after ADT is correlated with PIAS3, and pSTAT3 expression levels were increased after CRPC, which is consistent with our *in vitro* results (Supporting Information Fig S7).

### Gene profiling analysis using public database show increased CCL2 in human PCa tissues and androgen-deprived mouse prostates

In order to corroborate our findings with the link of AR silencing to CCL2 in other experimental settings, we analysed microarray studies deposited in the public NCBI database (Varambally et al, 2005); (Wang et al, 2007), we took advantage of these gene-profiling databases and found increased CCL2 expression in PCa tissues (Supporting Information Fig S8A**)**. Most importantly, increased expression of CCL2/CCR2 and EMT markers was observed in mouse prostates after castration (Supporting Information Fig S8B–H), suggesting that androgen deprivation in prostate glands elicits signalling pathways for CCL2/CCR2/EMT induction Therefore, the data of gene profiling analysis is consistent with our working hypothesis showing AR functions as a negative regulator of CCL2/CCR2/EMT signalling.

## DISCUSSION

Recent efforts have shed new light on molecular pathways linking CCL2 and PCa progression (Zhang et al, 2010a,2010b,2010c). It has been known that CCL2 promotes PCa progression via recruitment of macrophages into the PCa microenvironment and enhancing PCa cell growth and survival (Loberg et al, 2007). However, little is known about the mechanisms linking androgen/AR suppression and CCL2 induction in PCa cells. Our present study first established a previously unrecognized role of AR in negatively regulating CCL2 expression in PCa cells and TAMs, suggesting the current ADT only targeting androgen/AR in the prostate tumour microenvironment may help to create an immunosuppressive tumour microenvironment via induction of CCL2, which is similar to wound healing studies showing ARKO mice had an accelerated wound healing process (Lai et al, 2009).

By comparing AR roles in the wound healing process and PCa microenvironment, the interplay between AR silencing via siAR and induction of CCL2 may serve as a key step for initiating the infiltration of macrophages into PCa lesions. This emerging paradigm implicates that the current ADT with a single therapeutic approach via targeting androgen/AR in the PCa microenvironment may trigger unwanted pathways that promote macrophage infiltration, reprogram macrophages into TAMs with pro-tumour functions, and enhance EMT, all of which eventually result in increasing PCa cell migration/invasion via induced CCL2.

Our data showed that EMT is an important process involved in AR silencing-mediated/enhanced PCa invasion, suggesting a suppressive role for PCa AR in regulating EMT. Importantly, in our co-culture models, the crosstalk between macrophages and PCa cells also enhances signalling pathways that drive EMT in PCa cells upon AR silencing via siAR, indicating that this regulation points to macrophages as a key component of the PCa microenvironment that promotes EMT of PCa cells. We postulated that induction of Snail and MMP9 that orchestrated EMT programs in PCa cells during co-culture, could be triggered by macrophages and PCa AR silencing (Zhu & Kyprianou, 2010). Ultimately, our data support a model that AR silencing via siAR in PCa cells can trigger CCL2 induction and then reinforce the impacts of infiltrating TAMs on PCa cells, and foster PCa cell invasion with the initiation of EMT.

Based on our data, there is a close interplay between macrophages and PCa cells: AR silencing via siAR in both cell types leads to induction of CCL2. We therefore hypothesized that AR silencing via siAR in macrophages may also trigger CCL2 expression during the crosstalk with PCa cells. To study the *in vivo* role of AR in macrophages for PCa development and progression, we established the MARKO/TRAMP mouse model and found that the ablation of macrophage AR enhanced PCa development and metastatic potential with increased macrophage infiltration, CCL2 induction, STAT3 activation, and EMT. Interestingly, increased CCL2 and PCa metastasis was observed in TRAMP mice with AR ablation in either prostate epithelial cells or macrophages (Niu et al, 2008), supporting that CCL2 expression triggered by AR silencing in either cell type could be an initiating signal for later activation of the CCL2/STAT3/EMT signalling pathways.

Intriguingly, our data suggested that AR silencing-mediated CCL2 induction resulted in enhanced PCa migration/invasion, but AR silencing via siAR also reduced PCa cells growth, which is not in agreement with an early study showing CCL2 is a potent inducer of PCa cell proliferation (Loberg et al, 2006). It is possible that via modulation of EMT, this could explain the slower growth of AR silenced PCa cells since invasive tumour cells with EMT often manifested slow proliferation with lower expression of Ki67 and increased cell cycle inhibitor, p16/INK4A (Brabletz, 2012). This suggests that EMT and the growth capacity of PCa cells seem to be mutually exclusive. Our PCa mouse model clearly demonstrated that increased CCL2 and EMT markers in AR silenced PCa cells were associated with increased distant metastasis, in spite of reduced size of orthotopic AR silenced primary tumours. This suggests that the CCL2/EMT axis could be operative while AR in PCa cells was repressed by ADT to help form pre-metastatic PCa niches for further progression, which may eventually contribute to the failure of ADT.

Our recent work also showed that PCa patients receiving ADT had increased PCa stem/progenitor cell population, and found that AR might play a negative role in regulating this population (Lee et al, 2013), suggesting that ADT may preferentially promote the survival of PCa stem/progenitor cells via inhibiting androgen/AR function. Most importantly, our studies raise the possibility that targeting androgen/AR by ADT or siRNA may help to select PCa stem/progenitor cells via CCL2/EMT signalling pathways, since more and more evidence supports an interesting phenomenon that cancer cells that have undergone EMT often share similar characteristics with stem/progenitor cells (Gupta et al, 2009). Also, a recent study identified a novel role for CCL2 showing that CCL2 stimulates the self-renewal of stem/progenitor cells in breast cancer (Tsuyada et al, 2012). Therefore, this will be our future direction to investigate whether CCL2 promotes the selection of PCa stem/progenitor cells with inhibiting AR function or losing AR expression via an EMT-dependent pathway during ADT.

**Figure 10:**
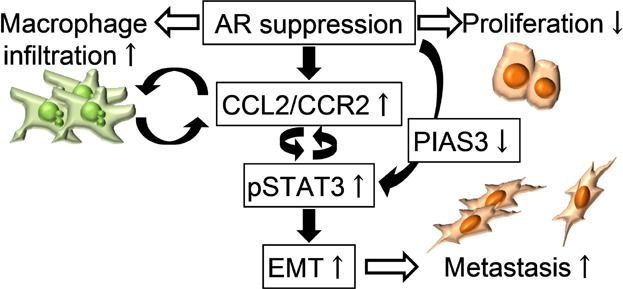
A working model of the crosstalk between macrophages and PCa cells that involve CCL2, STAT3 and EMT pathways in the androgen-deprived tumour microenvironment The data from this study support the model that AR suppression facilitates metastasis of PCa cells through CCL2/CCR2/STAT3 axis accompanied with increase of macrophage infiltration into PCa site.

Our findings also support a new role of AR silencing via siAR in mediating the induction of EMT via CCL2-STAT3 activation in the tumour microenvironment. This evidence is in accord with a previous study showing that constitutive STAT3 activation in normal prostate epithelial cells enhances EMT and cell motility (Azare et al, 2007). Consistent with this study, our *in vitro* and *in vivo* data demonstrated that targeting AR via siAR in PCa cells reduced PIAS3 expression that could possibly result in STAT3 activation-induced CCL2 expression, which might represent a key step to increase macrophage recruitment, as well as promote further STAT3 activation and EMT in PCa cells that ultimately enhanced PCa invasion at later stages. An early study showed that castration could elicit various leucocyte recruitments to PCa sites, which eventually resulted in the development of castration resistance through induction of lymphotoxin from B cells (Ammirante et al, 2010). Our findings resonate with this study, supporting a possible mechanism that current ADT in the PCa microenvironment may induce unwanted inflammation signals and further promote PCa progression. Most importantly, skeletal metastasis occurs in approximately 80% of patients with advanced PCa, and no curative therapies are available for metastatic CRPC to date (Denis, 1993; Rubin et al, 2000). Interestingly, it was previously demonstrated that CCL2 increased bone metastasis of PCa cells (Mizutani et al, 2009). Therefore, our findings established a novel link between targeting AR via siAR and the CCL2/CCR2-STAT3-EMT axis and provide new therapeutic targets to prevent potential PCa metastasis at later stages (Fig 10).

Finally, our analyses of the TMA collection of 73 specimens from prostatectomy confirmed the clinical significance of our findings identifying CCL2/STAT3/Snail as potential markers for PCa progression. In addition, valuable clinical results from the same patients before and after CRPC implicate that CCL2 could be also an important mediator for PCa progression, not only in hormone naïve PCa but also in CRPC, and potentially contribute to the development of CRPC. Most importantly, our pilot study using clinical samples is consistent with the gene profiling data of one elegant study of CRPC cells showing CCL2 is one of the AR repressed genes via the epigenetic modification with lysine specific demethylase (LSD1) (Cai et al, 2011). Therefore, it will be an interesting direction to investigate whether the induction of CCL2/CCR2-STAT3-EMT signals and the regulation of LSD1 function by AR silencing could support surviving PCa cells to advance into the castration-resistant stage.

Our study has identified the CCL2/CCR2-STAT3-EMT axis as potential new targets to improve the clinical outcome of PCa patients under ADT, and combination therapy of targeting AR and anti-CCL2/CCR2 (and also probably its downstream mediator, STAT3) might help us to better battle PCa at the castration resistant stage.

## MATERIALS AND METHODS

### Antibodies and chemicals

Anti-GAPDH (6c5), anti-β-actin (I-19) and anti-AR (N-20) antibodies were purchased from Santa Cruz Biotechnology. Anti-E-cadherin (MAB1838) antibody was from R&D systems. Anti-tSTAT3 (9132) and pSTAT3 (9131, T705) were from Cell Signaling. Anti-MMP9 (ab38898), anti-Snail (ab85931) and anti-PIAS3 (ab22856) antibodies were from Abcam, and anti-PSA antibody (A0562) was from DAKO. Anti-F4/80 antibody (123101) was from Biolegend, anti-CCL2 antibody (HPA019163) was from Sigma–Aldrich and Anti-CCL2 antibody (554661) for neutralization study was from BD Biosciences. Anti-CD68 antibody (MA1-80133) was from Thermo. The CCR2 antagonist (sc-202525) was from Santa Cruz Biotechnology, and the STAT3 inhibitor (AG490, 658401) was from Calbiochem.

### Cell culture and co-culture experiments

LNCaP cells and LAPC4 cells (androgen sensitive human PCa cell lines), and C4-2 cells (androgen-independent human PCa cell line), were maintained in RPMI-1640 medium with 5% (10% for LAPC4) foetal bovine serum and 1% penicillin/streptomycin. TRAMP-C1 cells (mouse PCa cell line), were maintained in DMEM with 10% foetal bovine serum, 1% penicillin/streptomycin and 0.005 mg/ml insulin (Invitrogen). THP-1 cells (human acute monocytic leukaemia cell line), were maintained in RPMI-1640 medium with 10% foetal bovine serum, 1% penicillin/streptomycin and 2-mercaptoethanol to a final concentration of 0.05 mM. RAW264.7 cells (mouse macrophage cell line), were maintained in RPMI-1640 medium with 10% foetal bovine serum, and 1% penicillin/streptomycin. All cell lines were obtained from the American Type Culture Collection. All cultures were grown in a humidified 5% CO_2_ environment at 37°C. Twenty-four-well transwell plates were used for co-culture experiments (BD Biosciences).

### Cell proliferation assay

C4-2 and TRAMP-C1 cells were seeded in six-well plate (10^5^ cells/well) and cultured for 24, 48 and 72 h. In co-culture experiments, C4-2 and LNCaP cells were seeded in 24-well transwell plates (0.4 μm 10^4^ cells/well) and insert wells including THP-1 cells (10^4^ cells/well) were put into each cancer cell seeded-well, and cultured for 24, 48 and 72 h with or without treatment. Cells were harvested and cell numbers were counted in triplicate using a haemocytometer.

### Cell migration assay

*In vitro* cell migration assay was performed using 24-well transwell inserts (8 μm for cancer cells and 5 μm for THP-1 cells) according to the manufacturer's instructions. C4-2, LNCaP and THP-1 cells (10^5^ cells/well) were seeded in the upper chamber of transwell plates and THP-1 cells (10^5^ cells/well for migration assay of cancer cells), C4-2 or LNCaP cells (10^5^ cells/well for migration assay of THP-1 cells) or control medium was added to the lower chamber. Cells were incubated for 24 h (for migration assay of cancer cells) or 16 h (for migration assay of THP-1 cells). The PCa cells migrated to the lower part of the membrane were stained and counted in six random fields, and migrated THP-1 cells into lower chamber were collected and centrifuged, and counted using a haemocytometer.

### Human cytokine antibody array and ELISA

CM was collected from THP-1, LNCaP, C4-2, or from co-cultures of LNCaP cells + THP-1 cells or C4-2 cells + THP-1 cells for 24 h. Relative amounts of cytokine levels were determined using Human Cytokine Array kit (Panel A, ARY005, R&D Systems) according to the manufacturer's instructions. CM collected from monocultures or co-cultures were also used for detection of CCL2 by human CCL2 ELISA kits (R&D Systems) according to the manufacturer's instructions.

### RNA extraction and quantitative real-time PCR analysis

Total RNA was isolated using Trizol reagent (Invitrogen) according to the manufacturer's instructions. One microgram of total RNA was subjected to reverse transcription using Superscript III transcriptase (Invitrogen). RT-PCR has been described previously (Zhang et al, 2006). Primers used were: CCL2 forward, 5′-GTC TCT GCC GCC CTT CTG TG-3′ and CCL2 reverse, 5′-GAC ACT TGC TGC TGG TGA TTC TTC-3′; CCR2 forward, 5′-CTG TCC ACA TCT CGT TCT CGG TTT A-3′ and CCR2 reverse, 5′-CCC AAA GAC CCA CTC ATT TGC AGC-3′; β-actin forward, 5′-TGT GCC CAT CTA GGA GGG GTA TGC-3′ and β-actin reverse, 5′-GGT ACA TGG TGG TGG CGC CAG ACA-3′. Quantitative real-time PCR (qRT-PCR) was conducted using a Bio-Rad CFX96 system with SYBR green to determine the level of mRNA expression of a gene of interest. Expression levels were normalized to the expression of β-actin RNA.

**The paper explained**

PROBLEM:

Increased inflammatory cells in PCa lesions have been observed after targeting AR by ADT. It has been demonstrated that the interaction of infiltrating macrophages and PCa cells mediated the hormone resistance of PCa cells. Recent studies have highlighted an important role of macrophages in promoting tumour growth and progression. However, whether AR suppression in PCa cells is the main driving force of PCa progression via increasing cytokine induction and macrophage recruitment remains unclear.

RESULTS:

In this work, we report that CCL2, a novel AR silencing-induced cytokine in PCa cells, is able to promote PCa cell invasion/metastasis through macrophage recruitment, STAT3 activation, and EMT when AR is functionally suppressed in PCa and macrophage cells during *in vitro* co-culture. Consistently, *in vivo* ablation of AR in myeloid or prostate cells promotes metastatic progression of PCa cells through induction of CCL2. Our study demonstrates that AR silencing in PCa cells prompts CCL2 expression via STAT3 activation by downregulation of a STAT3 protein inhibitor, PIAS3. The enhancement of the CCL2/STAT3/EMT axis by AR silencing in the tumour microenvironment could contribute to PCa progression.

IMPACT:

We identified CCL2 as an AR silencing-induced cytokine that enhances macrophage infiltration, activates STAT3, and induces EMT while prostate epithelial cells interact with macrophages during ADT. Our findings show CCL2 contributes critically to promote AR silenced PCa cell invasion/metastasis, which provides more insights into better therapeutic design of combined targeting of the AR and CCL2/CCR2 axis for preventing PCa progression led by CCL2.

### Western Blot Analysis

Cells were lysed in RIPA buffer (50 mM Tris–HCl/pH 7.4, 1% NP-40, 150 mM NaCl, 1 mM EDTA, 1 mM PMSF, 1 mM Na3VO4, 1 mM NaF, 1 mM okadaic acid and 1 mg/ml aprotinin, leupeptin and pepstatin). Individual samples (15–30 μg protein) were prepared for electrophoresis run on 8–12% SDS/PAGE gel and then transferred onto PVDF membranes (Millipore). After blocking the membranes with 5% fat free milk in TBST (50 mM Tris/pH 7.5, containing 0.15 M NaCl and 0.05% Tween-20) for 1 h at room temperature, the membranes were incubated with appropriate dilutions of specific primary antibodies overnight at 4°C. After washing, the blots were incubated with anti-rabbit, anti-mouse, or anti-goat IgG horseradish peroxidases for 1 h. The blots were developed in ECL mixture (Thermo Fisher Scientific Inc.).

### Zymography

The CM of C4-2 scr and siAR cells treated with CCL2ab was collected and activity of MMP9 was determined by zymography using 10% native polyacrylamide gels, as previously described (Henke et al, 2006; Sood et al, 2010). Activity was visualized as light staining bands on a dark background and normalized to the total amount of protein present in each sample as previously described (Deatrick et al, 2013; Henke et al, 2006; Sood et al, 2010).

### Generation of the MARKO/TRAMP Mice

We generated the MARKO/TRAMP mice by first mating fAR/X female mice (C57BL/6 background) with LyzCre^+/+^ male mice (C57BL/6 background) to produce the fAR/X-LyzCre^+/−^ female mice. We then mated fAR/X-LyzCre^+/−^ female mice with LyzCre^+/+^ male mice to generate fAR/X-LyzCre^+/+^ female mice. After this step, we also can get fAR/Y-LyzCre^+/+^ male (MARKO) mice. And then we mated fAR/X-LyzCre^+/+^ female mice with TRAMP male mice on a C57BL/6 background to generate MARKO/TRAMP male mice and WT/TRAMP littermates for our experiments. Floxed AR mice on a C57BL/6 background were generated by inserting *loxP* sites to flank exon 2 of *AR* gene (Yeh et al, 2002). TRAMP and LyzCre (C57BL/6 background) mice were purchased from The Jackson Laboratory. TRAMP and floxed AR alleles in tail genomic DNA of MARKO/TRAMP mice can be detected by polymerase chain reaction (PCR) as described previously (Zhang et al, 2006). Primers used for genotyping were: flox AR select, 5′-GTT GAT ACC TTA ACC TCT GC-3′ and flox AR 2-9, 5′-CTT ACA TGT ACT GTG AGA GG-3′; Lyz cre (WT), 5′-TTA CAG TCG GCC AGG CTG AC-3′, (cre), 5′-CCC AGA AAT GCC AGA TTA CG-3′ and (common), 5′-CTT GGG CTG CCA GAA TTT CTC-3′; TRAMP forward, 5′-TAC AAC TGC CAA CTG GGA TG-3′ and TRAMP reverse, 5′-CAG GCA CTC CTT TCA AGA CC-3′. Protocols for use of animals were in accordance with regulatory standards as approved by the University Committee on Animal Resources at the University of Rochester Medical Center.

### Orthotopic implantation

TRAMP-C1 cells were directly injected into the anterior prostates (AP) of athymic nude mice. After anaesthesia, the abdomens of 8–10-week-old athymic nude mice were surgically opened in sterile environments. TRAMP-C1 cells (2 × 10^6^ cells/AP) suspended in 10 μl of media mixed with 10 μl of Matrigel (BD Biosciences) were injected into both AP lobes by 30-gauge needle, and the abdomens were closed using silk sutures. Nude mice were treated with drugs by i.p. injection every other day from 2 weeks after tumour cell injection. One group was injected with TRAMP-C1 scramble cells and treated with vehicle (*n* = 9), two other groups were injected with TRAMP-C1 siAR cells. In these two groups, one group was treated with vehicle (*n* = 12), and the other group was treated with 50 µg/kg CCR2 antagonist (*n* = 17). Tumours were harvested at 20 days after starting treatment.

### HE Staining

The tissue sections were de-waxed and rehydrated routinely. The sections were stained in haematoxylin for 5 min, and washed in running tap water for 5 min. Then the sections were stained in eosin for 30 s, dehydrated, and mounted by routine methods. The representative fields were chosen to present in the figures.

### TUNEL assay

The tissue sections were de-waxed and rehydrated routinely and used for detection of apoptotic cells by *In Situ* Cell Death Detection Kit, Fluorescein (Roche) according to the manufacturer's instructions. The positive cells were counted in six random fields.

### Histology and IHC staining

Mouse prostate tissues were fixed in 10% v/v formaldehyde in PBS, embedded in paraffin, and cut into 5 μm sections. Six prostate tissues from each group were randomly picked for staining. Prostate sections were deparaffinized in xylene solution and rehydrated using gradient ethanol concentrations. IHC staining was performed as described previously (Wu et al, 2007). Commercially available human prostate TMA (PR243a and PR956) was purchased from US Biomax Inc. PSA values and survival data of some patients in these TMAs are available. TMA sections (4 μm) were immunohistochemically labeled, using the specific primary antibodies to CCL2 and CD68. Seventy-three PCa specimens obtained by radical prostatectomy performed at the University of Rochester Medical Center were also immunohistochemically labeled, using the specific primary antibodies to snail, CCL2, and pSTAT3. German Immunoreactive Score (0–12) was calculated, separately in benign and malignant glands, by multiplying the percentage of immunoreactive cells (0% = 0; 1–10% = 1; 11–50% = 2; 51–80% = 3; 81–100% = 4) by the staining intensity (negative = 0; weak = 1; moderate = 2; strong = 3). Scores were considered negative (0–1), weakly positive (2–4), moderately positive (6–8), and strongly positive (9–12). The macrophage infiltration number was calculated with CD68 or F4/80 positive cells. The average number of macrophages in an ocular measuring field at 400× magnification was used for statistic analysis.

### Human prostate cancer biopsy specimens

Human prostate needle biopsy specimens were obtained from the Department of Urology, University of Kanazawa, after receiving approval from the Institutional Review Board of Kanazawa University. Four pairs of needle biopsy specimens at diagnosis and then later after developing CRPC from the same patient and 1 needle biopsy specimen from a CRPC patient were analysed with IHC staining. These samples were neither from transurethral resection nor autopsies, but needle biopsy. The same analytical methods used in TMA were used in analyses of these biopsy specimens.

### Statistics

The data values were presented as the mean ± SEM. *p*-values were determined by unpaired Student's *t*-test using commercially available software (Prism 5) unless special methods were mentioned. *p* < 0.05 was considered statistically significant.

## Supplementary material

As a service to our authors and readers, this journal provides supporting information supplied by the authors. Such materials are peer reviewed and may be re-organized for online delivery, but are not copy-edited or typeset. Technical support issues arising from supporting information (other than missing files) should be addressed to the authors.
