# Supplementary material for: Targeting the androgen receptor with siRNA promotes prostate cancer metastasis through enhanced macrophage recruitment via CCL2/CCR2-induced STAT3 activation
Source: EMBO Mol Med. 2013 Aug 27;5(9):1383–401. doi: 10.1002/emmm.201202367 (PMC3799493; doi:10.1002/emmm.201202367)
Supplement: Supplementary file 1 [file emmm0005-1383-SD1.pdf]

EMBO
Molecular Medicine

Targeting the androgen receptor promotes prostate cancer metastasis through enhanced macrophage recruitment via CCL2/CCR2-induced STAT3 activation

Kouji Izumi, Lei-Ya Fang, Atsushi Mizokami, Mikio Namiki, Lei Li, Wen-Jye Lin and Chawnshang Chang

Corresponding author: Chawnshang Chang, University of Rochester

Review timeline:

Submission date:	04 December 2012
Editorial Decision:	11 December 2012
Manuscript transferred:	12 December 2012
Editorial Decision:	31 January 2013
Revision received:	30 April 2013
Editorial Decision:	28 May 2013
Revision received:	21 June 2013
Accepted:	26 June 2013

Transaction Report:

(Note: With the exception of the correction of typographical or spelling errors that could be a source of ambiguity, letters and reports are not edited. The original formatting of letters and referee reports may not be reflected in this compilation.)

Editors: Thomas Schwarz-Romond, Roberto Buccione

1st Editorial Decision 11 December 2012

Thank you very much for submitting your research manuscript for consideration to The EMBO Journal editorial office.
We assessed your paper very carefully, based on relevant literature as well as discussing potential suitability from the perspective of The EMBO Journal within the entire editorial team.

We appreciate the novel link between therapeutic androgen receptor deprivation and CCL2-up regulation that subsequently contributes to increased PCa cell migration/invasion via EMT and STAT3-signal activation. We also realize that these events are corroborated in genetic models, explored for therapeutic intervention and nicely correlated with patient expression data and tumor biopsies. From a more conceptual perspective however, all these events appear not without earlier precedence. The major value of your study thus seems the careful delineation of androgen deprivation-induced molecular events, respective their potential therapeutic exploitation. Given such a translational perspective, I am afraid we had to judge your study not suited according to the strong conceptual demands of The EMBO Journal.

Given the clear translational implications however, I took the liberty to inquire with a colleague from our sister journal EMBO Molecular Medicine (EMM) (http://www.embomolmed.org/view/0/index.html). I am thus happy to communicate that their scientific editor Roberto Buccione would be prepared to expose your study to peer-review.

I thus kindly ask you to submit/contact Roberto directly (r.buccione@embomolmed.org) to enable efficient proceedings.

I am sorry that I am unable to reach a more positive conclusion from the perspective of The EMBO Journal, but I hope that EMM might turn out to be a suitable alternative for presentation of your results.

2nd Editorial Decision 31 January 2013

Thank you for the submission of your manuscript to EMBO Molecular Medicine. We have now heard back from the three Reviewers whom we asked to evaluate your manuscript.

You will see that all three Reviewers are supportive of your work and underline its interest. However, a few significant concerns were raised that require your action and thus publication of the paper cannot be considered at this stage. I feel it unnecessary to report each point in detail here as they are clearly stated by the Reviewers, but I will mention a few salient items.

Reviewer 1 mentions two technical/data presentation issues that need to be solved. S/he also requires further in vitro and in vivo experimentation to better understand the mechanism of STAT3 activation through PIAS3.

Reviewer 2 lists a number of points that require your action. A number of these are important items of clarification that need to be carefully and precisely addressed within the manuscript. Others are experimental issues that require further investigation, For instance, Reviewer 2 mentions the need for additional cases to convincingly demonstrate the clinical relevance of your tenet that CCL2 correlates with macrophage recruitment in castration-resistant prostate cancer. S/he also notes that it is important to differentiate between pro- and anti-inflammatory macrophages in your experiments

Reviewer 3, while generally supportive of your work, contends that androgen receptor silencing is not necessarily equivalent to the clinical setting and suggests experimental approaches to help improve this aspect. I strongly advise that you experimentally address this issue in your manuscript to improve impact and overall clinical relevance, an important factor for EMBO Molecular Medicine.

In conclusion, although publication of the paper cannot be considered at this stage, we would consider a suitably revised version, with the understanding that the Reviewers' concerns must be fully addressed with additional experimental evidence where required.

Please note that it is EMBO Molecular Medicine policy to allow a single round of revision only and that, therefore, acceptance or rejection of the manuscript will depend on the completeness of your responses included in the next, final version of the manuscript.

As you know, EMBO Molecular Medicine has a "scooping protection" policy, whereby similar findings that are published by others during review or revision are not a criterion for rejection. However, I do ask you to get in touch with us after three months if you have not completed your revision, to update us on the status. Please also contact us as soon as possible if similar work is published elsewhere.

I look forward to receiving your revised manuscript as soon as possible.

***** Reviewer's comments *****

Referee #1 (Comments on Novelty/Model System):

Overall, this work is interesting and has novel aspects. Quality of data is also generally good. However, some improvement is required:
Fig.3E: MMP signals are not convincing: Zymography recommended
Fig.4K/L: the Stat3 actvating mechanism through downregulation of PIAS3 needs further elucidation in vitro and in vivo; SOCS3 and TcPTP45 should be analyzed and included

Fig.8B: AR as well as pStat3 and PIAS3 staining need to be included

Referee #2 (Comments on Novelty/Model System):

A central premise for this article is that when PCa cells die during ADT there is "wound-healing associated inflammation" and recruitment of macrophages to the prostate (and to the bone, assuming there is bone metastasis). It is important to point out that when PCa cells die during ADT by apoptosis, it is not associated with an inflammatory response. It may be misleading to compare cancer to a "wound" and cancer therapy to "wound-healing", because in real patients an inflammatory response is rare, and if it is observed in animal studies, it could be an artifact.

During ADT, AR+ cells are eliminated, tumor burden is reduced, and AR- cells may be selected. These AR- cells are known to express stem-ness features (Collins AT, Cancer Res, 2005), including wound-healing signatures, EMT, STAT3, etc. It makes sense that these AR- cells could also interact with macrophages (and mesenchymal stem cells, and other stromal cells such as osteoblasts, etc) and could be activated by inflammatory factors. Depending on the hypothesis you wish to test, do you think that your experiments may also be true for AR- cells (e.g., PC-3 or DU-145 cells) rather than AR ablated cells? In which case, promotion of prostate cancer metastasis through CCL2 induction by STAT3 activation would be AR independent!

Referee #2 (Remarks):

Thank you for the opportunity to review this very interesting manuscript. Many people believe that the microenvironment plays an important role in the pathogenesis of prostate cancer. There is also evidence that stromal cells and paracrine factors produced by stromal cells may be involved in the modulation of pro-inflammatory and angiogenesis factors. This study connects a potentially crucial interaction between macrophages through CCL2 induction and the androgen receptor. It has implications on the nature of castration-resistant prostate cancer and how we can potentially improve androgen deprivation therapy by combining it with CCL2-targeted therapy.

I have several comments, which I hope may further enhance the quality of this manuscript.

1. I agree that combining ADT (which eliminates AR+ PCa cells) with CCL2/CCR2 axis-targeted therapy may provide improved control of the primary cancer and delay metastasis. However, I am not convinced about the rationale for the mechanism of action and by the experiments to support a potentially erroneous or irrelevant hypothesis.

2. A central premise for this article is that when PCa cells die during ADT there is "wound-healing associated inflammation" and recruitment of macrophages to the prostate (and to the bone, assuming there is bone metastasis). It is important to point out that when PCa cells die during ADT by apoptosis, it is not associated with an inflammatory response. It may be misleading to compare cancer to a "wound" and cancer therapy to "wound-healing", because in real patients an inflammatory response is rare, and if it is observed in animal studies, it could be an artifact.

3. During ADT, AR+ cells are eliminated, tumor burden is reduced, and AR- cells may be selected. These AR- cells are known to express stem-ness features (Collins AT, Cancer Res, 2005), including wound-healing signatures, EMT, STAT3, etc. It makes sense that these AR- cells could also interact with macrophages (and mesenchymal stem cells, and other stromal cells such as osteoblasts, etc) and could be activated by inflammatory factors. Depending on the hypothesis you wish to test, do you think that your experiments may also be true for AR- cells (e.g., PC-3 or DU-145 cells) rather than AR ablated cells? In which case, promotion of prostate cancer metastasis through CCL2 induction by STAT3 activation would be AR independent!

4. Suppose an inflammatory response is observed and macrophages are present, how do you determine if it is cause, effect, or incidental with respect to AR function? In all likelihood, an inflammatory response is just as detrimental in castration-resistant as in androgen-dependent prostate cancer. I suspect that in castration-resistant prostate cancer, other pathways besides the AR pathway may play an increasingly important role in the pathogenesis of this disease.

5. Because there are both proinflammatory (M1) and anti-inflammatory (M2) macrophages that may be involved, you need to differentiate them in your experiments.

6. Importantly, we need more cases for Figure 8, which provides clinically-relevant evidence, if not proof, for the whole idea of this article that CCL2 correlated with macrophage recruitment in the setting of castration-resistant prostate cancer. For example, it is not true that the number of macrophages (Figure 8C) correlated with CCL2 expression (Figure 8B), especially for cases A and B that turned out to be just the opposite of what you would have predicted.

I recommend approval of this manuscript for publication, after the above comments have been addressed.

Referee #3 (Remarks):

In this work, androgen receptor is silenced by siRNA in prostate cancer cells and macrophages and authors show that silencing of AR led to increased CCL2 expression, increased PCa migration and macrophage recruitment and this is through CCL2/CCR-STAT3 axis. This observation is corroborated by an elegant experiment with mice lacking in macrophage AR and other experiments with human TMA and gene expression analysis.

Major comment
1) What happens downstream of AR silencing is well supported in many cell line and mouse model systems and analysis of primary tumor specimens strengthens the hypothesis. However, the major problem is that the model systems differ from what happens in the clinical setting. Human patients who are diagnosed with metastatic prostate cancer are treated with castration therapy (or androgen deprivation therapy) +/- antiandrogen (such as bicalutamide). Silencing of AR with knockdown of AR may not be equivalent to the clinical scenario and the result of silencing of AR might be more complete or less complete loss of AR function.

Authors should test the effect of androgen depletion (by using charcoal-stripped serum media) in addition to silencing of AR. Many of the experiment in Figure 1-4, especially those involving androgen responsive cell lines like LNCaP and LAPC4 cells, should be repeated by using androgen depleted media compared to control. For C4-2, it is expected that androgen depletion may not have any effect, since AR function in these cells may not respond to androgen depletion. A novel potent anti-androgen like enzalutamide (MCV3100) should be used to inhibit AR function in C4-2 cells.

Minor comment
1) The phrase "AR silenced-induced" or "AR silenced-mediated" throughout the manuscript should be changed to "AR silencing-induced" or "AR silencing-mediated".

1st Revision - authors' response 30 April 2013

Referee #1 (Comments on Novelty/Model System):

Overall, this work is interesting and has novel aspects. Quality of data is also generally good.

However, some improvement is required:

1. Fig.3E: MMP signals are not convincing: Zymography recommended.

Ans: Thank you for your suggestion. We have repeated the experiment with the MMP9 zymography, and the result is consistent with our previous data shown in Fig. 3E. We have included this data in Fig. 3E.

2. Fig.4K/L: the Stat3 activating mechanism through downregulation of PIAS3 needs further elucidation in vitro and in vivo; SOCS3 and TcPTP45 should be analyzed and included.

Ans: Thank you for your comments. We have examined SOCS3 and TcPTP45 in various PCa cells (scramble and siAR), and found (Fig. 1) (figure removed from this Peer Review Process file) that the expression of TcPTP45 and SOCS3 was not reduced by knockdown of AR, which is distinct from the downregulation of PIAS3 expression in PCa-siAR cells. This data suggests that AR may predominantly regulate the expression of PIAS3 proteins in PCa cells, consistent with one previous report showing DHT can induce PIAS3 expression in LNCaP cells (*Biochem Biophys Res Commun 2000;278:9-13*).

As for the STAT3 activating mechanism, one elegant paper by Michael Karin's group first showed increased pSTAT3 in xenograft prostate tumors after castration (Nature 2010;464:302-5), suggesting STAT3 activation could be an important support for the survival of prostate cancer cells during androgen deprivation. In this paper, they presented a detailed mechanism that tumor-infiltrating B cells induce STAT3 activation in xenograft tumors after castration. Interestingly, we observed STAT3 activation by AR silencing in PCa cells (siAR) without the involvement of infiltrating inflammatory cells, suggesting that there could be an intrinsic mechanism involved in this STAT3 activation inside AR silencing PCa cells. Our main hypothesis is that PCa-siAR cells may downregulate protein inhibitors of STAT3 and relieve their repression of STAT3 activity. We agree with the reviewer's comment that we should have explored the detailed mechanisms of PIAS3 downregulation in PCa-siAR cells. We have tried a few times by stably transfecting PIAS3 into PCa-siAR cells. However, we failed. There is a technical challenge in achieving this goal due to the growth arrest of PCa-siAR cells, as shown in Fig. 4D. After one or two passages, we often lost the majority of PCa-siAR cells. It is impossible to further isolate stable PIAS3-expressing cells under antibiotic selection. Therefore, we adapted an alternative approach using a chemical STAT3 inhibitor (AG490) to block STAT3 activation (Fig. 4J of the manuscript), and demonstrated that STAT3 activation is important for CCL2/EMT induction.

Most importantly, in order to correlate the downregulation of PIAS3 with STAT3 activation in PCa-siAR cells, we performed IHC of PIAS3 in TRAMP-C1 sc and siAR xenograft tumors, and found that only very few TRAMP-C1 siAR tumor cells contain PIAS3 protein (Fig. 2) (figure removed from this Peer Review Process file). Consistently, we showed (Fig. 6h of the manuscript) increased pSTAT3/CCL2/EMT protein levels in TRAMP-C1 siAR tumors, which correlated well with PIAS3 downregulation in the same TRAMP-C1 siAR tumor cells.

3. Fig.8B: AR as well as pStat3 and PIAS3 staining need to be included

Ans: Thank you for your comments. We decided to move this figure into the supplementary figures and replace with our 2nd TMA analysis. Due to the difficultly of acquiring more paired specimens from same patients since PCa patients do not necessarily have another biopsy procedure after being diagnosed with developing castration resistance. Previously, we were fortunate to have 4 volunteer patients who were willing to go through another biopsy procedure to support our study. Indeed, we have performed IHC of AR, pSTAT3, and PIAS3 on these paired prostate cancer specimens. Generally, the expression of AR is correlated with PIAS3, and found decreased after ADT. In contrast, the expression of pSTAT3 is increased in CRPC patients (Supplementary Fig. 6), supporting a possible scenario that the inhibition of the AR activity by ADT reduced the expression of PIAS3 protein, and then relieve its inhibition of STAT3 function, which may have negative impacts on ADT therapy. However, it remains to be determined in our future study whether this pathway would contribute to the development of CRPC since the growth of PCa-siAR cells is significantly reduced. This suggests that this cell models is still dependent on the functional activity of AR.

More importantly, in order to confirm our findings, we repeated IHC of CCL2/pSTAT3/Snail on another prostate tissue microarray (revised Fig. 8). The results suggest that patients with increased Snail expression had poor recurrence-free survival, which is significantly correlated with CCL2, supporting a role for the CCL2/pSTAT3/EMT(Snail) axis during PCa progression.

© EMBO

Referee #2 (Comments on Novelty/Model System):

1. A central premise for this article is that when PCa cells die during ADT there is "wound-healing associated inflammation" and recruitment of macrophages to the prostate (and to the bone, assuming there is bone metastasis). It is important to point out that when PCa cells die during ADT by apoptosis, it is not associated with an inflammatory response. It may be misleading to compare cancer to a "wound" and cancer therapy to "wound-healing", because in real patients an inflammatory response is rare, and if it is observed in animal studies, it could be an artifact.

Ans: Thank you for your comments. The citation of the wound healing and cancer progression in the introduction of our manuscript was to establish the common feature of AR in mediating inflammatory responses via macrophages between the wound healing process and prostate cancer progression, since both tend to have an immunosuppressive microenvironment. We did not intend to tell readers that ADT would trigger wound healing-like inflammation when PCa cells undergo apoptosis. Most importantly, evidence has suggested that ADT does induce infiltration of immune cells (T cells and macrophages) into prostate (*J Immunol Methods 2009;348:9-17, Proc Natl Acad Sci U S A 2001;98:14565-70*). It is not necessarily the case for these immune cells to proceed via a wound healing-like process during ADT. Interestingly, the previous paper concluded that macrophages seem to favor the development of advanced PCa since they found a high density of CD68(+) macrophages was related to an increased risk of biochemical recurrence, which is consistent with our working hypothesis in this regulation. Therefore, we believe this is not an artifact that was only observed in mouse models.

2. During ADT, AR+ cells are eliminated, tumor burden is reduced, and AR- cells may be selected. These AR- cells are known to express stemness features (Collins AT, Cancer Res, 2005), including wound-healing signatures, EMT, STAT3, etc. It makes sense that these AR- cells could also interact with macrophages (and mesenchymal stem cells, and other stromal cells such as osteoblasts, etc) and could be activated by inflammatory factors. Depending on the hypothesis you wish to test, do you think that your experiments may also be true for AR- cells (e.g., PC-3 or DU-145 cells) rather than AR ablated cells? In which case, promotion of prostate cancer metastasis through CCL2 induction by STAT3 activation would be AR independent!

Ans: Thank you for your comments. We are pleased to know our data with PCa-siAR cells are consistent with other studies using AR-null PCa cells. Nevertheless, PCa-siAR cells in our study were not completely without AR expression (Fig. 1E and 2F). Our finding suggest that inhibition of AR function may trigger this regulation, and it doesn't have to be completely null with AR. Importantly, Pienta's group has demonstrated that PC3 cells with stable CCL2 expression increased the growth of transplanted xenografts, enhanced bone metastasis, and increased the accumulation of macrophages *in vivo (Pienta's paper: Neoplasia 2009;11:1235-42*). Their findings suggest that CCL2 in PC3 cells increases tumor growth and bone metastasis through recruitment of macrophages, and also implicate that CCL2 promotes PCa metastasis by increasing macrophage recruitment in AR-null PC3 cells in an AR-independent manner. Whether STAT3 activity was increased in PC3-CCL2 PCa cells remains unclear, since Pienta did not further characterize this cell model with STAT3 pathway. We postulated that STAT3 could likely be activated in PC3-CCL2 cells since CCL2 has been shown to be induced in cancer-associated fibroblasts (*Cancer Res 2012;72:2768-79*)

Referee #2 (Remarks):

Thank you for the opportunity to review this very interesting manuscript. Many people believe that the microenvironment plays an important role in the pathogenesis of prostate cancer. There is also evidence that stromal cells and paracrine factors produced by stromal cells may be involved in the modulation of pro-inflammatory and angiogenesis factors. This study connects a potentially crucial interaction between macrophages through CCL2 induction and the androgen receptor. It has implications on the nature of castration-resistant prostate cancer and how we can potentially improve androgen deprivation therapy by combining it with CCL2-targeted therapy.
I have several comments, which I hope may further enhance the quality of this manuscript.
1/2. I agree that combining ADT (which eliminates AR+ PCa cells) with CCL2/CCR2 axis-targeted therapy may provide improved control of the primary cancer and delay metastasis. However, I am not convinced about the rationale for the mechanism of action and by the experiments to support a

potentially erroneous or irrelevant hypothesis. A central premise for this article is that when PCa cells die during ADT there is "wound-healing associated inflammation" and recruitment of macrophages to the prostate (and to the bone, assuming there is bone metastasis). It is important to point out that when PCa cells die during ADT by apoptosis, it is not associated with an inflammatory response. It may be misleading to compare cancer to a "wound" and cancer therapy to "wound-healing", because in real patients an inflammatory response is rare, and if it is observed in animal studies, it could be an artifact.

Ans: Thank you for your comment. As we comment a similar question raised by the reviewer 2 in the above section, the citation of the wound healing and cancer progression in the introduction of our manuscript was to establish the common feature of AR in mediating inflammatory responses via macrophages between wound healing process and prostate cancer progression, since both tend to have an immunosuppressive microenvironment. We did not intend to tell readers that ADT must trigger wound healing-like inflammation when prostate cancer cells undergo apoptosis. Our wound healing model only implicated that ARKO may tend to create an immunosuppressive environment that favors for wound healing process, which first established a role for AR to modulate inflammatory responses via macrophages, and supports a possible role for AR in regulating inflammatory cytokines in the tumor microenvironment. That's the focus of our study. We didn't address ADT-induced PCa cell death that has to be associated with the wound-healing process. However, there are some interesting references suggesting that wound healing process resembles the metastatic cancer progression (Chang, H. Y. et al. Robustness, scalability and integration of a wound-response gene expression signature in predicting breast cancer survival. *Proc Natl Acad Sci U S A 2005;102:3738-43*), since extra cellular matrix remodelling and cell migration are common features shared by wound healing and tumor progression (*Nat Rev Cancer 2005;5:244*). In reference to that, we believe there may be some similar function of AR in wound healing and prostate tumor microenvironment in terms of the regulation of inflammatory signaling pathways. But we want to emphasize that this paper did not intend to establish the link between wound healing and prostate cancer progression.
Most importantly, literatures shows ADT or experimental castration-induced massive inflammatory infiltration in the prostate (*J Immunol Methods 2009;348:9-17, Proc Natl Acad Sci U S A 2001;98:14565-70, Nature 2010;464:302-5*). These studies were examining either human or mouse prostate tissues after ADT or surgical castration, and clearly found increased inflammatory infiltrates in the prostate. Therefore, it could exclude the possibility of a potential artifact of seeing inflammation after ADT only observed in mouse models.

3. During ADT, AR^+ cells are eliminated, tumor burden is reduced, and AR^- cells may be selected. These AR^- cells are known to express stem-ness features (Collins AT, Cancer Res, 2005), including wound-healing signatures, EMT, STAT3, etc. It makes sense that these AR- cells could also interact with macrophages (and mesenchymal stem cells, and other stromal cells such as osteoblasts, etc) and could be activated by inflammatory factors. Depending on the hypothesis you wish to test, do you think that your experiments may also be true for AR- cells (e.g., PC-3 or DU-145 cells) rather than AR ablated cells? In which case, promotion of prostate cancer metastasis through CCL2 induction by STAT3 activation would be AR independent!
Ans: This question is the same as the question above. Please refer the answer to the 2nd comment on novel/model system of reviewer 2.

4. Suppose an inflammatory response is observed and macrophages are present, how do you determine if it is cause, effect, or incidental with respect to AR function? In all likelihood, an inflammatory response is just as detrimental in castration-resistant as in androgen-dependent prostate cancer. I suspect that in castration-resistant prostate cancer, other pathways besides the AR pathway may play an increasingly important role in the pathogenesis of this disease.

Ans: Thank you for your comment. It has been shown macrophage-derived inflammatory cytokines such as IL-1beta can trigger AR activation via MEKK1 by inhibiting the corepressor function and contribute to castration resistance, since AR can respond to anti-androgens as an agonist (*Cell 2006;124:615-29*), which represents the first potential model of macrophage-mediated castration resistance. This important study has provided solid evidence showing the crosstalk between macrophage and PCa cells would potentially bring negative impacts on ADT when AR no longer responds to anti-androgens. The PCa-associated inflammation could be mediated by multi-factors as mentioned by reviewers. The point we want to address here is the role of AR suppression by ADT

and its downstream signaling pathways that may be associated with inflammation since ADT-induced inflammation in prostate has been well documented as described above (*J Immunol Methods 2009;348:9-17, Proc Natl Acad Sci U S A 2001;98:14565-70*). We established co-culture and animal models, and identified CCL2 is a key player in mediating AR silencing-mediated inflammatory response. Therefore, our study suggests that CCL2 may be the unwanted signal during ADT since it may promote PCa survival, invasion, and metastasis via STAT3-EMT pathways. We conclude that the inflammation could possibly be the consequential effects of ADT in the tumor microenvironment via induction of CCL2.

5. Because there are both proinflammatory (M1) and anti-inflammatory (M2) macrophages that may be involved, you need to differentiate them in your experiments.

Ans: Thank you for your suggestion. We believe the macrophage cell line in our study could be skewing toward M2-like polarization. One interesting study has shown THP-1 cells behave as M2-like macrophages, and promotes the growth of colon cancer cells (*Oncogene 2009;28:3892–902*). Therefore, we adapted the same cell lines for establishing our co-culture model. Importantly, in order to see the polarization of THP-1 cells, we have examined several M2 markers in THP-1 cells after co-culture with PCa cells, since Pienta's group showed CCL2 promote M2 polarization of macrophages (*J Biol Chem 2009;284:34342-54*). We observed AR silencing in THP-1 cells significantly induced several M2 markers during co-culture with C4-2 cells (Supplementary Figure 2), suggesting a potential scenario that AR-silencing may promote THP-1 cells skewing more toward M2 type macrophages via CCL2, which could explain why ARKD THP-1 cells (*in vitro*) or MARKO macrophages (*in vivo*) can act like tumor-associated macrophages and promote tumor progression.

6. Importantly, we need more cases for Figure 8, which provides clinically-relevant evidence, if not proof, for the whole idea of this article that CCL2 correlated with macrophage recruitment in the setting of castration-resistant prostate cancer. For example, it is not true that the number of macrophages (Figure 8C) correlated with CCL2 expression (Figure 8B), especially for cases A and B that turned out to be just the opposite of what you would have predicted. I recommend approval of this manuscript for publication, after the above comments have been addressed.

Ans: Thank you for your suggestion. We truly understand the weakness of this data. We decided to move this figure into the supplementary figures and replace this figure with our 2nd TMA analysis due to the difficultly of acquiring more paired specimens from the same patients. As you know, the biopsy of prostate tissue after patients develop CRPC is usually not performed clinically because the pathological diagnosis is no longer needed for the decision of new treatment for CRPC, unless there is a strong indication that patients may have neuroendocrine differentiation. Also, the biopsy procedure is often associated with huge costs, adverse events, hospitalization, pain, and anesthesia. Therefore, it is very difficult to get paired samples from the same patient during diagnosis and after developing CRPC. As described above, we were very fortunate to have 4 volunteer patients who were willing to go through another biopsy procedure that allowed us to examine whether CCL2 and downstream molecules are associated with the development of CRPC. This is the reason why we could not acquire more samples in order to get solid conclusions. The most important limitation on having needle biopsy samples is that the volume is very small, only a few slides can be kept for our study. As we described in manuscript about the value of these paired samples, it has been known that autopsy samples of CRPC patients and transurethral resection samples of CRPC patients with benign prostate hyperplasia are often subjected to autolysis and heat damage, which may prevent accurately assessing gene expression. In contrast, we believe our biopsy samples should maintain their original status and characteristic. Despite of only 4 paired samples, we have performed IHC of AR, pSTAT3, and PIAS3 on these paired prostate cancer specimens. Generally, the expression of AR is correlated with PIAS3, found decreased after ADT. In contrast, the expression of pSTAT3 is increased in CRPC patients (Supplementary Fig. 6), supporting a possible scenario that the inhibition of the AR activity by ADT reduced the expression of PIAS3 protein, and then relieved its inhibition on STAT3 function, which may have negative impacts on ADT therapy. However, it remains to be determined in our future study whether this pathway would contribute to the development of CRPC, since the growth of AR silencing (to mimic inhibiting AR function by ADT) PCa cells is significantly inhibited (Fig. 4D), which means the growth of PCa cells is still dependent on DHT/AR signaling in our culture condition. Therefore, we decided to move this data into supplementary figure, and performed another TMAs in order to confirm whether our working model

with CCL2/STAT3/EMT(snail) is associated with PCa progression. Consistently, we found the expression of CCL2/STAT3/ Snail is associated with each other and patients with this increased expression of this CCL2 axis would have shorter recurrence-free survival, suggesting this axis could have prognostic value in predicting PCa behaviors.

Referee #3 (Remarks):

In this work, androgen receptor is silenced by siRNA in prostate cancer cells and macrophages and authors show that silencing of AR led to increased CCL2 expression, increased PCa migration and macrophage recruitment and this is through CCL2/CCR-STAT3 axis. This observation is corroborated by an elegant experiment with mice lacking in macrophage AR and other experiments with human TMA and gene expression analysis.

Major comment

What happens downstream of AR silencing is well supported in many cell line and mouse model systems and analysis of primary tumor specimens strengthens the hypothesis. However, the major problem is that the model systems differ from what happens in the clinical setting. Human patients who are diagnosed with metastatic prostate cancer are treated with castration therapy (or androgen deprivation therapy) +/- antiandrogen (such as bicalutamide).

Ans: Thank you for your comments. We agree with the reviewer's comment regarding ADT for metastatic prostate cancer, which was originally established based on Charles Huggins' pioneer study. More importantly, many literatures show that ADT (including combined androgen blockade) is used for locally advanced PCa as well as metastatic PCa (*Lancet 2011;378:2104–11*). In addition, ADT has been used as an initial treatment for localized prostate cancer (*J Urol 2006;176:S47–49*). Therefore, our study could be a potential working model for the progression of localized PCa.

Silencing of AR with knockdown of AR may not be equivalent to the clinical scenario and the result of silencing of AR might be more complete or less complete loss of AR function. Authors should test the effect of androgen depletion (by using charcoal-stripped serum media) in addition to silencing of AR. Many of the experiment in Figure 1-4, especially those involving androgen responsive cell lines like LNCaP and LAPC4 cells, should be repeated by using androgen depleted media compared to control. For C4-2, it is expected that androgen depletion may not have any effect, since AR function in these cells may not respond to androgen depletion. A novel potent anti-androgen like enzalutamide (MDV3100) should be used to inhibit AR function in C4-2 cells.

Ans: Thank you for your comments.
1. The major reason why we adapt silencing of AR instead of antiandrogens or androgen deprivation (media with charcoal-stripped FCS) is because Steven Balk's group has proved intratumor de novo androgen synthesis in PCa cells, such as VCaP and C4-2 cells (*Cancer Res 2011;71:6503-13*), suggesting PCa cells may activate AR by their own newly synthesized androgen while cultured in the media with charcoal-stripped FCS. Therefore, we decided to adapt AR silencing as an alternative approach to block AR function in PCa cells instead of using traditional anti-androgens and androgen-deprived medium, since PCa cells are capable of synthesizing androgen. Therefore, traditional androgen depletion may not effectively block AR function. To confirm intratumoral androgen synthesis, we also have performed GC/MS analysis to quantify androgen levels in PCa cells that were cultured in the media with charcoal-stripped FCS. Our unpublished observation shows that most PCa cell lines (either androgen responsive or androgen unresponsive) are able to synthesize androgens at different levels.
2. As for MDV3100 experiments, our concurrent study has demonstrated that MDV3100 and Casodex will trigger the TGFβ/smad3/MMP9 signaling pathway and promote prostate cancer cell invasion (Differential Androgen Deprivation Therapies Promote or Suppress Prostate Cancer Metastasis. Tzu-Hua Lin et. al., paper in submission to American Journal of Pathology). We show the unpublished data (Fig. 1 of Tzu-Hua Lin's paper) here to support our paper (figure removed from this Peer Review Process file).

The figure demonstrated that antiandrogens (Casodex or MDV3100) inhibit the cell growth, but promoted cell invasion. This data is consistent with this revised study showing suppression of AR, by either AR silencing or antiandrogens, promote PCa cell migration/invasion. Further data will be

provided upon request if reviewers wish to understand our concurrent study regarding anti-androgens.

Minor comment
The phrase "AR silenced-induced" or "AR silenced-mediated" throughout the manuscript should be changed to "AR silencing-induced" or "AR silencing-mediated".

Ans: Thank you for your suggestion. We have revised that in our manuscript.

3rd Editorial Decision 28 May 2013

Thank you for the submission of your revised manuscript to EMBO Molecular Medicine. We have now heard back from the three Reviewers whom we asked to evaluate your manuscript.

You will see that while all three Reviewers are supportive of you work, Reviewer 2 has a remaining concern that I would ask you to act upon before we can accept your manuscript for publication.

Reviewer 2 notes that your findings are also compatible with an alternative hypothesis/explanation. Given that to formally test this hypothesis would be beyond the scope of the current manuscript, I am prepared to make a rapid editorial decision on your manuscript, provided you take this alternative interpretation into consideration in your discussion.

Please send me your revised manuscript (with description of the changes applied) as quickly as possible and in any case within two weeks.
I look forward to receiving your re-revised manuscript as soon as possible.

***** Reviewer's comments *****

Referee #1 (Comments on Novelty/Model System):

The revision is nicely done. and the concerns raised addressed.

Referee #2 (Comments on Novelty/Model System):

The authors performed some very elegant experiments that demonstrated suppression of AR function promoted prostate cancer metastasis through CCL2 induction via STAT3 activation. Importantly, they showed that the microenvironment also played a role in this process by recruitment of macrophages.

The authors are correct about a well-known clinical observation: "therapeutic approach that is solely based on targeting AR may be insufficient to control PCa cells." However, the hypothesis that "suppressing AR function may elicit unwanted signals that may favor the progression of surviving PCa cells to the advanced stage" is based on an experimental observation: "genetic ablation of AR in prostate epithelial cells promoted the development of invasive PCa tumors." After all, experiments should be done to test a hypothesis, not to form a hypothesis.

Perhaps another related and relevant clinical observation in this regard concerns patients with nonfunctioning AR (as in eunuchs), who are actually less disposed to develop prostate cancer (Wu CP, 1991).

I believe the authors should test an alternative hypothesis: AR- prostate cancer stem cells may be selected during ADT, are activated by the same inflammatory process through their interaction with macrophages, as well as with other cells such as CAF or mesenchymal stem cells (Brennen WN, 2013; Anton K, 2012), and are involved with CCL2/STAT3/EMT, snail/Notch1 signaling pathways (Tsuyada A, 2013).

So the critical question is whether the same findings of prostate cancer progression through CCL2 induction via STAT3 activation are AR-dependent or independent. If the latter is true, then AR may not be causal in these events. For example, inflammatory responses and wound healing are associated with progressive cancer with high malignant potential, but they do so in the absence of AR in many other cancer types (Beachy PA, 2004;Brannon AR, 2010).

Interestingly, CCL2/STAT3/Snail was also found to be a valuable prognostic marker in hormone naïve prostate cancers with AR+ cells in the TMA collection of 73 prostatectomy specimens (most of which have not undergo ADT).

The finding that "AR functions as a negative regulator of CCL2/EMT signaling" is compatible with our understanding that AR is a differentiating factor in the prostate! "Progression of surviving PCa cells to the advanced stage" and to castration-resistant prostate cancer may indicate selection of AR- prostate CSCs rather than targeting of AR+ differentiated cells during ADT. The authors have clearly shown that the surviving PCa cells or AR- prostate CSCs express stem-ness markers, activate stem-ness signaling pathways, and interact with the microenvironment, all of which are compatible with the alternative hypothesis, as mentioned above.

Referee #3 (Remarks):

Authors had a reasonable response to my comments and provided additional data that have been submitted elsewhere for publication. I have no further comments.

2nd Revision - authors' response 21 June 2013

The revised manuscript originally entitled "Targeting the androgen receptor promotes prostate cancer metastasis through enhanced macrophage recruitment via CCL2/CCR2-induced STAT3 activation (EMM-2012-02367-V3)" is respectfully submitted for publication in EMBO Molecular Medicine. We revised the title of our manuscript according to your suggestion. Most importantly, we have revised Fig. 4L and upload this figure after you found an issue after image check. In addition, we realigned Fig. 6 C and D, since we found there is an overlapped area between the tumor photos (C) and the quantitation plot (D) during final check. We believe that our manuscript is now suitable for publication in EMBO Molecular Medicine.
Please let me know if you need any further information. Thank you for your editorial decision and we look forward to your rapid response.
