# Supplementary material for: Targeting the androgen receptor with siRNA promotes prostate cancer metastasis through enhanced macrophage recruitment via CCL2/CCR2-induced STAT3 activation
Source: EMBO Mol Med. 2013 Aug 27;5(9):1383–401. doi: 10.1002/emmm.201202367 (PMC3799493; doi:10.1002/emmm.201202367)

Supplementary information

Targeting androgen receptor promotes prostate cancer metastasis through CCL2 induction via STAT3 activation

Kouji Izumi, Lei-Ya Fang, Atsushi Mizokami, Mikio Namiki, Lei Li, Wen-Jye Lin and Chawnshang Chang

Table of contents:

Supplementary figure legends

Supplementary figures

Figure S1: Cytokine array analysis of the CM isolated from C4-2 scr and siAR cells.

Figure S2: qPCR analysis of M2 markers expression in THP-1 cells.

Figure S3: Western blot analysis of CCL2, EMT markers, and AR in parental LNCaP and LAPC4 cells after treated with CM of THP-1 scr and siAR, or co-cultured with THP-1 scr and siAR cells for 24 h.

Figure S4: Western blot analysis of CCL2, EMT markers, and AR in monoculture LNCaP and LAPC4 cells (scr and siAR).

Figure S5: IHC analysis of PIAS3 in TRAMP-C1 xenograft tumors (scr and siAR).

Supplementary Figure S6: The clinical information of prostate biopsy samples at diagnosis or CRPC stage.

Supplementary Figure S7: IHC analysis of prostate biopsy samples at diagnosis or CRPC stage.

Supplementary Figure S8: Gene profiling analyses using public database show increased CCL2 in human PCa tissues and androgen-deprived mouse prostates.

Supplementary Figure legends

Figure S1: Cytokine array analysis of the CM isolated from C4-2 scr and siAR cells. CM were collected after 24 h incubation (*left*). Increased CCL2 expression was observed in CM of C4-2 siAR cells (yellow square). The array map is shown at right. Ref=reference spot, Neg=negative control.

Figure S2: qPCR analysis of M2 markers expression in THP-1 cells. CCL22, MRC1, IL-10 and Arg1 expression in THP-1 scr or THP-1 siAR cells with or without co-cultured with C4-2 cells are shown.

Figure S3: Western blot of CCL2, EMT markers, and AR in parental LNCaP and LAPC4 cells after treated with CM of THP-1 scr and siAR, or co-cultured with THP-1 scr and siAR cells for 24 h. Similar regulation with CCL2, EMT markers, and AR in C4-2 cells was noted in LNCaP (*left*) and LAPC4 (*right*) cells co-cultured with THP-1 siAR cells.

Figure S4: Western blot analysis of CCL2, EMT markers, and AR in monoculture LNCaP and LAPC4 cells (scr and siAR). Similar regulation with CCL2, EMT markers, and AR in C4-2 scr and siAR cells was noted in LNCaP scr and siAR (*left*) and LAPC4 scr and siAR (*right*) cells.

Figure S5: IHC analysis of PIAS3 in TRAMP-C1 xenograft tumors (scr and siAR). The strong staining of PIAS3 in sections of TRAMP-C1 scr tumors is noted (left). In contrast, only marginal PIAS3 immunoreactivity in TRAMP-C1 siAR tumors is observed (right), suggesting AR silencing in TRAMP-C1 cells reduces the expression of PIAS3 protein. The strong PIAS3 staining in the benign control prostate gland indicates that benign prostate in TRAMP-C1 siAR tumors has not lost PIAS3. Black arrows indicate PIAS3-positive tumor cells. **T**: tumor; **B**: benign gland of anterior prostate.

Figure S6: The clinical information of prostate biopsy samples at diagnosis and CRPC stage. A. The patient's information from biopsy samples is shown. PSA values at diagnosis and at CRPC stage were statistically analyzed with paired one-tailed t test. TNM=TNM classification by UICC 1997, GS=Gleason score, Dx=diagnosis, Re-Bx=re-biopsy, and N/A=not available. **B.** The number of $CD68^+$ positive macrophages in prostate biopsy samples collected from patients during diagnosis or developing CRPC stage (*left*). The representative picture shows $CD68^+$ cells (arrows) in Case E at CRPC stage (*right*).

Figure S7: IHC analysis of prostate biopsy samples at diagnosis and CRPC stage. IHC of CCL2, AR, PIAS3, and pSTAT3 in prostate biopsy samples at diagnosis and at CRPC stage are shown, neg=negative; mod=moderate; str=strong. The specimen of PIAS3 in Case C was not available.

Figure S8: Gene profiling analyses using public database show increased CCL2 in human PCa tissues and androgen-deprived mouse prostates. A. GDS1439 database and **B-H.** GDS2562 database were obtained from the NCBI Gene Expression Omnibus (GEO) website. *Bars*, Mean±SEM.

Supplementary Fig. S1

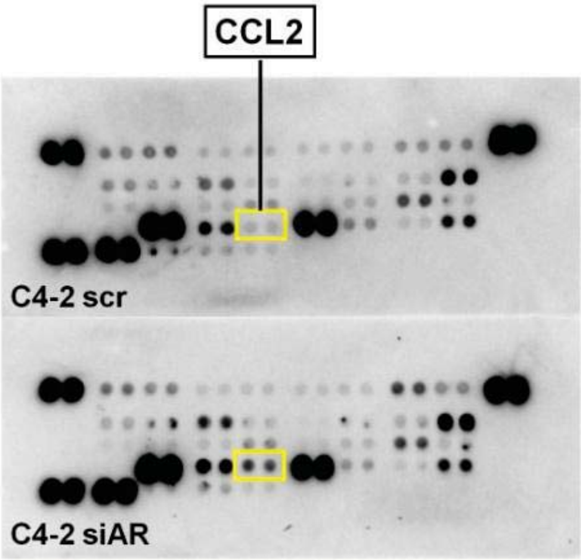

Ref	C5a	CD 154	G-CSF	GM-CSF	CXCL 1	CCL1	CD54	IFN-γ	Ref
	IL-1α	IL-1β	IL-1ra	IL-2	IL-4	IL-5	IL-6	IL-8	
	IL-10	IL-12p70	IL-13	IL-16	IL-17	IL-17E	IL-23	IL-27	
	IL-32α	CXCL 10	CXCL 11	CCL2	MIF	CCL3	CCL4	PAI-1	
Ref	CCL5	SDF-1	TNF-α	TREM -1					Neg

Supplementary Fig. S2

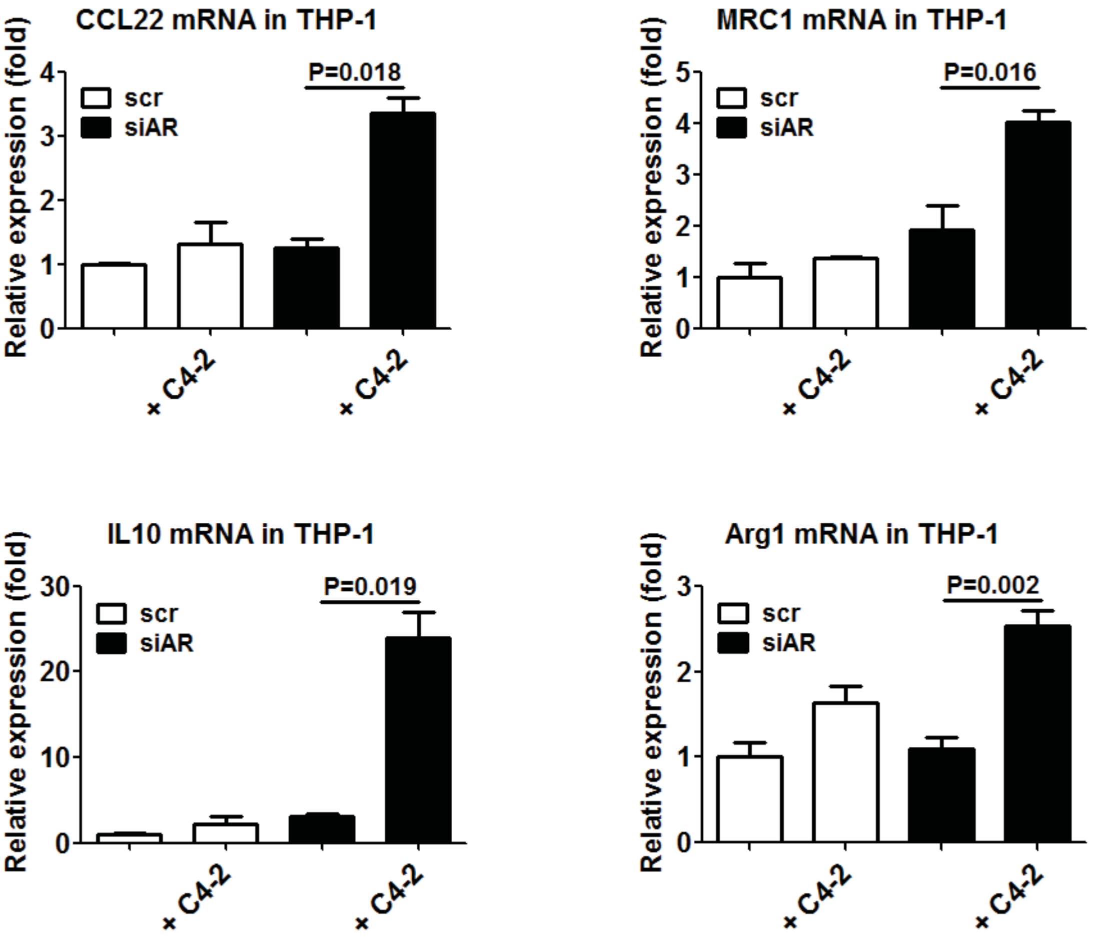

Supplementary Fig. S3

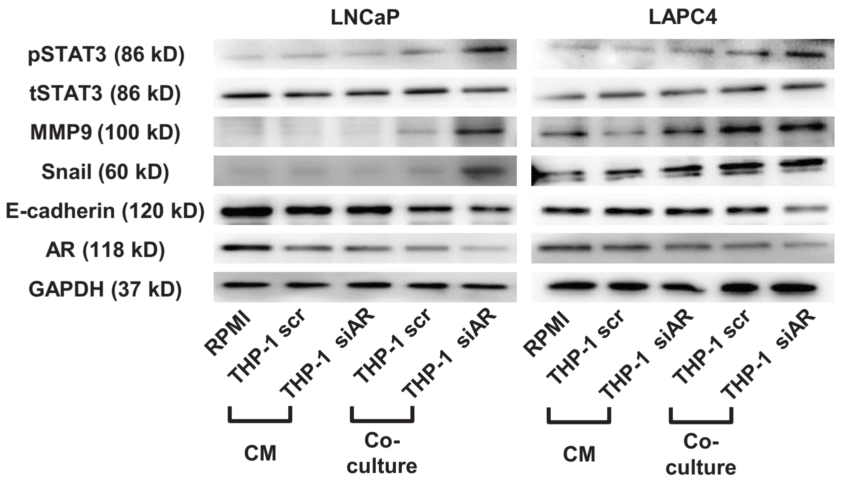

Supplementary Fig. S4

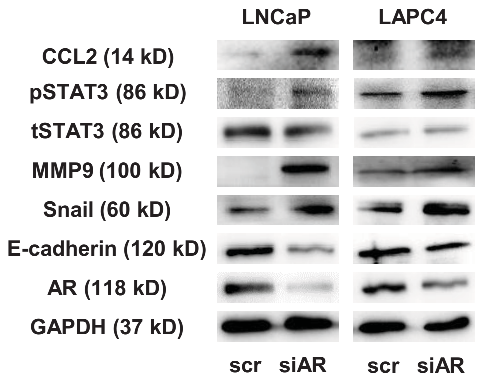

Supplementary Fig. S5

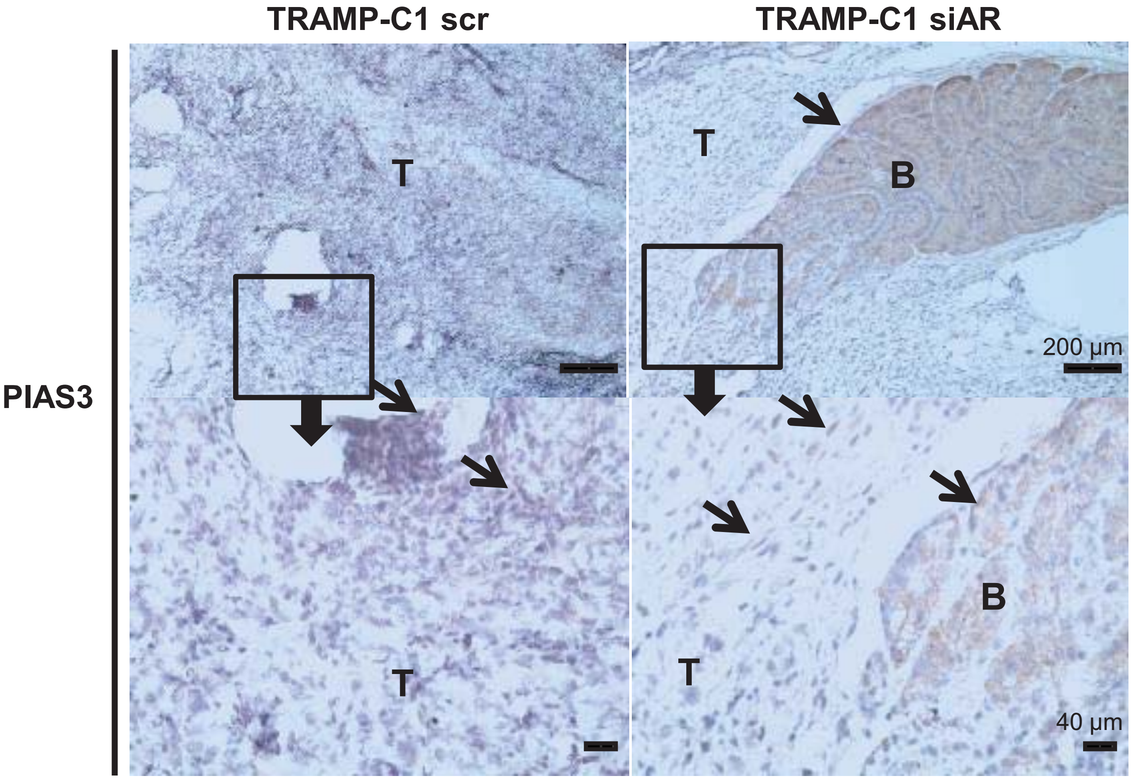

Supplementary Fig. S6

A

		Case A	Case B	Case C	Case D	Case E
Age (year)	at Dx	53	64	72	73	N/A
	at Re-Bx	57	68	76	77	80
PSA (ng/ml)	at Dx	46.4	196.6	71	246	N/A
	at Re-Bx*	0.017	11.326	0.194	198.079	185.25
TNM	at Dx	T3aN1M0	T4N1M0	T3aN0M0	T4N0M1b	N/A
GS	at Dx	3+4	5+5	4+4	4+5	N/A

*P=0.038

B

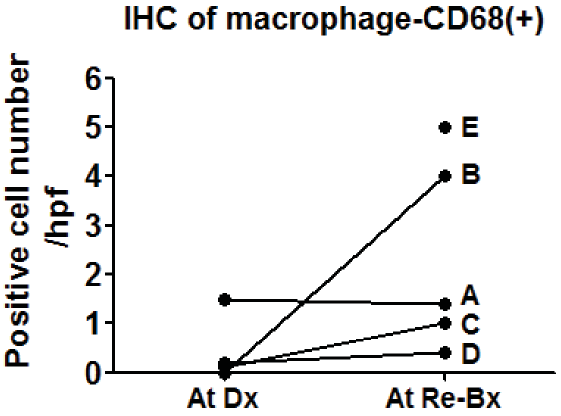

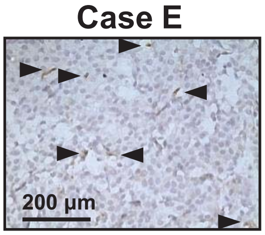

Supplementary Fig. S7

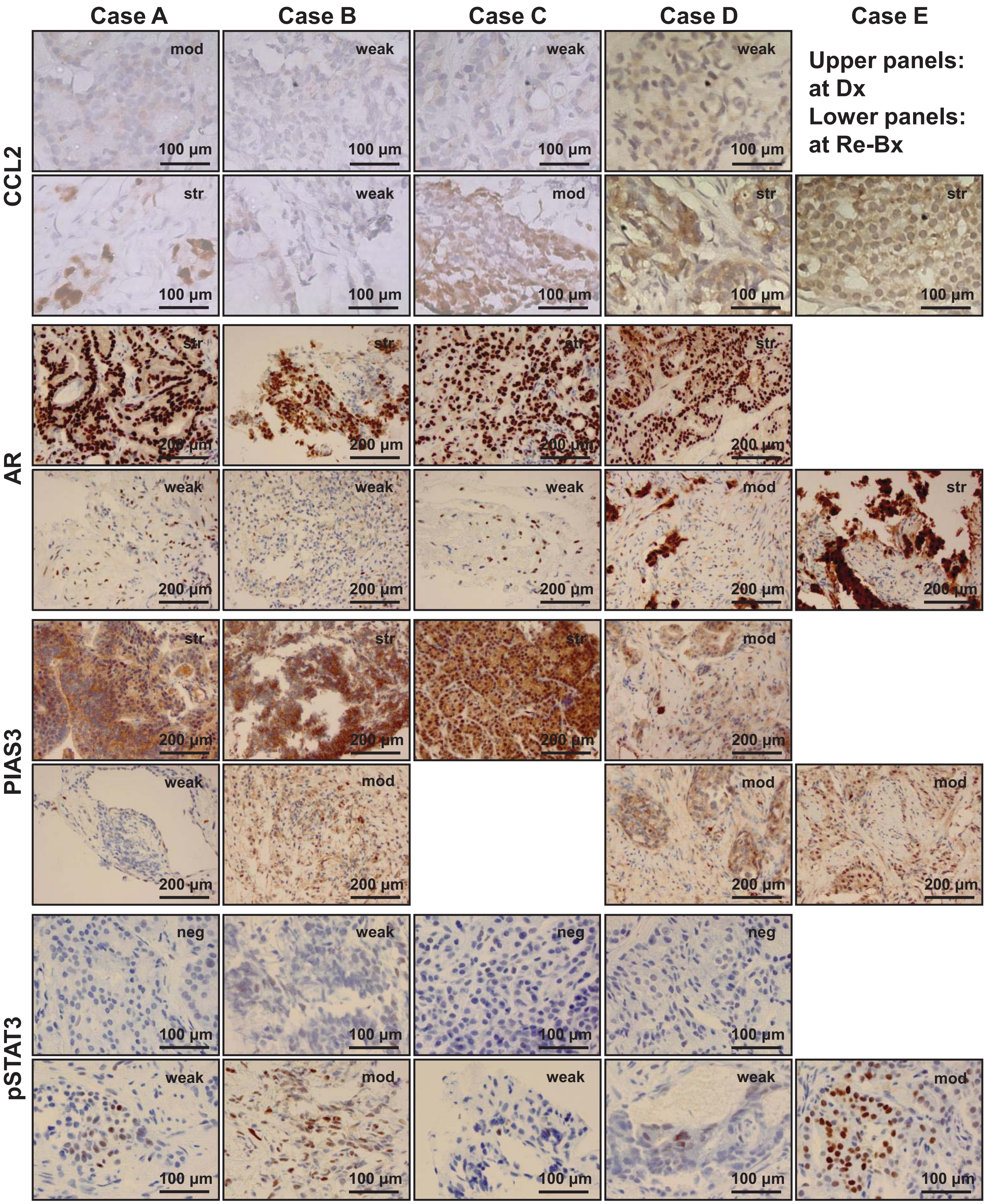

Supplementary Fig. S8

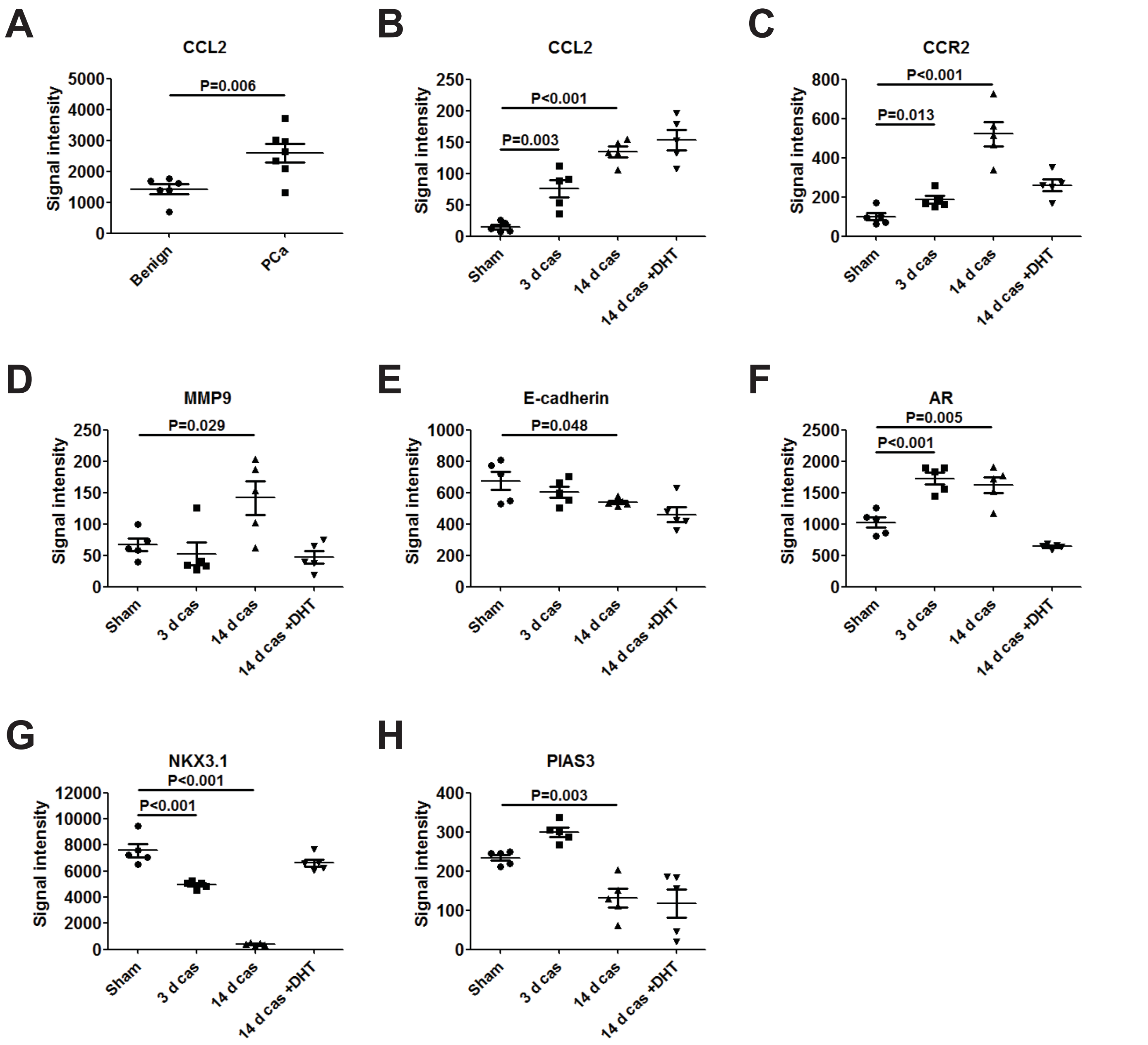

Supplement: Supplementary file 2 [file emmm0005-1383-SD2.pdf]
